# Dark Triad traits and workplace bullying: a systematic review and meta-analysis of personality, power, and psychosocial safety

**DOI:** 10.3389/fpsyg.2026.1738277

**Published:** 2026-03-04

**Authors:** Sophia Xin Sui, Sajida Malik, Niko Tiliopoulos, Lei Yu

**Affiliations:** 1 School of Psychology, Faculty of Health and Medical Science, University of Adelaide, Adelaide, SA, Australia; 2 School of Health and Biomedical Sciences, RMIT University, Melbourne, VIC, Australia; 3 Shandong University, Weihai, Shandong, China

**Keywords:** abusive supervision, Dark Triad, Machiavellianism, meta-analysis, narcissism, organizational psychology, perpetration, personality traits

## Abstract

**Background:**

Workplace bullying is a widespread and serious public health concern, affecting a substantial proportion of workers globally. While its consequences for mental and physical health are well established, consistent personality-based predictors of perpetration remain unclear. The Dark Triad personality traits, including Machiavellianism, narcissism, and psychopathy, are conceptually linked to interpersonal aggression but prior evidence is fragmented.

**Methods:**

We conducted a systematic review and meta-analysis following Preferred Reporting Items for Systematic Reviews and Meta-Analyses 2020 guidelines (Open Science Framework, DOI: 10.17605/OSF.IO/FWQ73). PubMed, PsycINFO, Embase, Scopus, and Web of Science were searched to 2 September 2025. Eligible studies reported associations between Dark Triad traits and workplace bullying perpetration among working-age participants. Random-effects models using Fisher’s z-transformed correlations generated pooled effect sizes. Risk of bias was assessed using an adapted Joanna Briggs Institute checklist, and certainty of evidence graded with Grading of Recommendations, Assessment, Development and Evaluations.

**Findings:**

Of 89 records screened, 16 met inclusion criteria, and nine contributed to meta-analysis. All traits were positively associated with bullying perpetration. Psychopathy showed the strongest pooled correlation (*r* = 0.53 [95% CI 0.28–0.71]), followed by narcissism (*r* = 0.40 [95% CI 0.26–0.52]) and Machiavellianism (*r* = 0.35 [95% CI 0.13–0.53]). Heterogeneity was substantial (*I*^2^ > 90%). Narrative synthesis indicated contextual moderators including organizational climate, justice perceptions, and identity threat—shaped trait expression.

**Interpretation:**

Machiavellianism, narcissism, and psychopathy are robust predictors of workplace bullying perpetration, with psychopathy most strongly implicated. Preventive strategies should integrate personality-informed leadership development with organizational safeguards. Workplace bullying should be prioritized as an occupational hazard and public health issue.

**Systematic review registration:**

https://osf.io/tvfkc/overview.

## Introduction

The International Labour Organization (ILO) estimates that more than 20% of workers globally experience workplace harassment or bullying during their careers, making it a major public health issue (International Labour, O. Lloyd’s Register, F. Gallup, 2022). Workplace bullying, defined as repeated and unreasonable behavior that creates risks to health and safety, has substantial consequences for both individuals and society (Safe Work, 2025). Victims face increased risks of psychiatric morbidity, including stress-related illness, anxiety, depression, burnout, and suicidal ideation, as well as physical health risks such as cardiovascular disease (Goh et al., 2022). Organizations incur significant costs through absenteeism, staff turnover, and lost productivity, further underscoring the societal burden of bullying (Malola et al., 2024).

Research on counterproductive work behavior has often focused on broad organizational deviance, such as theft or sabotage (Zappalà et al., 2021). Bullying, however, is distinct in that it represents interpersonal aggression directed at specific individuals (Notelaers et al., 2018). In addition to organizational and job-related antecedents, individual differences may shape who is more likely to engage in persistent interpersonal mistreatment, particularly in hierarchical work environments (Pauksztat and Salin, 2019). Among personality frameworks, the Dark Triad trait, comprised Machiavellianism, narcissism, and psychopathy, capture socially aversive dispositions characterized by manipulation, entitlement, and callousness (Čavlovič and Jug, 2025; Horsten et al., 2023). Conceptually, these traits are aligned with a willingness to dominate, exploit, and disregard others, making them plausible antecedents of bullying perpetration and abusive supervision (Özer and Escartín, 2023; Wai and Tiliopoulos, 2012).

Empirical findings on the link between Dark Triad traits and bullying have been inconsistent. Some studies identify strong associations (Dåderman and Ragnestål-Impola, 2019; Fernández-del-Río et al., 2022; Pilch and Turska, 2015), while others report weaker or null effects, particularly for narcissism (Dåderman and Ragnestål-Impola, 2019). Prior syntheses have combined victimization with perpetration or aggregated interpersonal aggression with organizational deviance (Hershcovis, 2011; Özer and Escartín, 2023). To our knowledge, no systematic review or meta-analysis has focused exclusively on bullying perpetration as a distinct outcome. Addressing this gap is crucial for advancing theory, informing organizational practice, and guiding public health strategies.

Within personality psychology, dark personality traits refer to a cluster of socially aversive dispositions characterized by interpersonal antagonism, manipulation, entitlement, and callousness. Individuals high on these traits tend to prioritize self-interest over collective goals, exhibit low concern for others’ welfare, and are more willing to exploit power asymmetries for personal gain (Pilch, 2020). Such characteristics are theoretically relevant to workplace bullying, which involves repeated interpersonal mistreatment often enacted within hierarchical or resource-dependent organizational contexts (Almeida et al., 2022).

Several theoretical models have been proposed to conceptualize dark personality. The Dark Core or D-factor framework posits a common underlying tendency toward maximizing personal utility at others’ expense as the shared core of socially aversive traits (Hilbig et al., 2021). More commonly applied in organizational research is the Dark Triad model, comprising Machiavellianism, narcissism, and psychopathy, which differentiates strategic manipulation, grandiosity and entitlement, and callous–impulsive tendencies, respectively (Schyns et al., 2019). An extension of this framework, the Dark Tetrad, incorporates everyday sadism, reflecting enjoyment derived from others’ suffering (Kay et al., 2025). These models vary in scope and granularity but overlap conceptually in their emphasis on antagonistic and exploitative interpersonal orientations.

The present review focuses on the Dark Triad model for both theoretical and methodological reasons. First, Machiavellianism, narcissism, and psychopathy represent the most extensively studied dark traits within organizational and occupational psychology, particularly in relation to leadership, counterproductive work behavior, and interpersonal aggression (Maftei et al., 2022). Second, the majority of empirical studies examining workplace bullying perpetration operationalize dark personality using Dark Triad–based measures, enabling meaningful synthesis and comparability across studies. While emerging evidence links everyday sadism to workplace aggression, the relative scarcity and heterogeneity of such studies currently limit the feasibility of quantitative synthesis. Accordingly, focusing on the Dark Triad allows for a theoretically coherent and methodologically robust meta-analysis while maintaining relevance to the existing workplace bullying literature.

Hence, we conducted a systematic review and meta-analysis to synthesize evidence on the association between Dark Triad personality traits and workplace bullying perpetration. The objectives of this study were to: (1) pool effect sizes for each trait and for the composite Dark Triad; (2) compare effects across outcome subtypes (bullying, abusive supervision, interpersonal deviance); and (3) synthesize contextual moderators across occupational settings. By focusing on perpetration, this study clarifies the role of aversive personality traits in workplace bullying and provides actionable insights for both organizational policy and public health intervention.

## Methods

This systematic review and meta-analysis was conducted and reported in accordance with the Preferred Reporting Items for Systematic Reviews and Meta-Analyses (PRISMA) 2020 guidelines (Page et al., 2021), which represent the current international standard for transparent reporting of systematic reviews and meta-analyses. The protocol for this systematic review was prospectively registered on the Open Science Framework (Sui, 2025). The full search protocol and extraction form are available in Appendix 1 to enhance transparency.

### Search strategy and study selection

PubMed, PsycINFO, Embase, Scopus, and Web of Science were searched from inception to 2 September 2025. We combined controlled vocabulary and free-text terms related to Dark Triad traits and workplace bullying, including synonyms for each trait and multiple operationalizations of bullying (e.g., abusive supervision, harassment, workplace aggression). Reference lists of eligible studies were screened manually, and citation chaining was used to capture additional records. Expert consultation was undertaken prior to finalizing the search strategy. This involved consultation with a health sciences academic librarian and two researchers with expertise in occupational psychology and workplace bullying, who reviewed the draft search strings, controlled vocabulary terms, and a set of sentinel papers to assess search sensitivity and coverage. Feedback from this process informed refinements to trait-related and outcome-related keywords, including alternative operationalizations of workplace bullying (e.g., abusive supervision and interpersonal counterproductive work behavior).

### Eligibility criteria

Studies were included if they: (1) reported quantitative associations between one or more Dark Triad traits (Machiavellianism, narcissism, psychopathy, or composite indices) and workplace bullying perpetration; (2) involved working-age participants in occupational settings; and (3) provided sufficient data to calculate or extract effect sizes. We excluded reviews, non-English papers, and studies focusing solely on victimization. To ensure our analysis focused on direct, predictive associations, studies that examined Dark Triad traits only as mediators or moderators were excluded from the meta-analysis but included in the narrative analysis.

### Screening and data extraction

Records were screened using Rayyan, a web-based platform designed to support systematic review screening, by two reviewers (SS and LY), who independently assessed titles, abstracts, and full texts for eligibility. Titles and abstracts were reviewed first, followed by full texts of potentially eligible articles. Disagreements were resolved through discussion or third-party arbitration (SM). Reasons for exclusion at the full-text stage were documented systematically (Appendix Table S1). Extracted data (Appendix Tables S2a,b) included study characteristics (country, year, sample size, occupational sector), trait measures [e.g., Short Dark Triad (SD3), Dirty Dozen], bullying outcomes (e.g., Negative Acts Questionnaire, Counterproductive Work Behavior) – Individual (CWB-I) indices, effect estimates, funding, and conflicts of interest. Where data were missing or unclear, authors were contacted for clarification. The extraction process was piloted to ensure consistency across reviewers.

### Risk of bias assessment

Risk of bias was assessed using an adapted Joanna Briggs Institute (JBI) checklist (Joanna Briggs, 2017; Moola et al., 2020) for analytical cross-sectional studies, modified for occupational-personality research to cover: sampling adequacy; validity and reliability of trait and bullying-perpetration measures (including alignment with construct definitions); handling of confounders; appropriateness of analytic methods; treatment of missing data; mitigation of common-method bias (e.g., multi-source or time-lagged designs); and transparency of reporting/ethics. The checklist evaluates key domains including sampling adequacy, validity and reliability of exposure and outcome measures, identification and control of confounding variables, appropriateness of statistical analyses, and transparency of reporting. Given the predominance of occupational and personality-based survey research, additional attention was paid to common-method bias, reliance on self-report measures, and temporal separation of variables where applicable. Overall risk-of-bias judgements were derived using prespecified criteria across domains. Two reviewers assessed each domain independently and resolved disagreements by consensus after a calibration exercise. Overall ratings followed a prespecified rule: “Low risk” only if all critical domains were adequate; “High” if ≥3 critical domains were inadequate or any fatal flaw was present; otherwise “Some concerns.” All included studies were rated “Some concerns.” Full domain-level judgements are provided in Appendix Table S3.

### Outcomes and synthesis

All statistical analyses were performed in R (Version 4.4.1; R Core Team, 2024) using the ‘metafor’ package. We provide the meta-analytic dataset Appendix Table S4 to enable independent re-analysis; code is available on request. The primary outcome was the correlation between Dark Triad traits and workplace bullying perpetration. When multiple measures were reported, we prioritized validated scales of perpetration. Effect sizes were standardized to correlation coefficients using Fisher’s z transformation. Random-effects models were applied using restricted maximum likelihood in R. Heterogeneity was assessed with *Q*, *I*^2^, and τ^2^ statistics, which tests whether observed variability exceeds that expected by chance; the *I*^2^ statistic, which estimates the proportion of total variance attributable to between-study heterogeneity; and τ^2^, which represents the estimated between-study variance in random-effects models. Subgroup analyses examined outcome subtypes (bullying perpetration, abusive supervision, interpersonal deviance), trait facets, and measurement instruments. Where data permitted, exploratory subgroup and sensitivity analyses examined whether pooled associations differed according to the Dark Triad measurement instrument used (e.g., Short Dark Triad, Dirty Dozen). Sensitivity analyses also excluded high-risk studies or outliers to test robustness. Funnel plots and Egger’s tests assessed small-study effects, with recognition of limited power when *k* < 10. Certainty of evidence was appraised using the Grading of Recommendations, Assessment, Development and Evaluations approach (Guyatt et al., 2008), considering study quality, consistency, directness, precision, and publication bias.

## Results

### Study selection and characteristics

**Figure 1:**
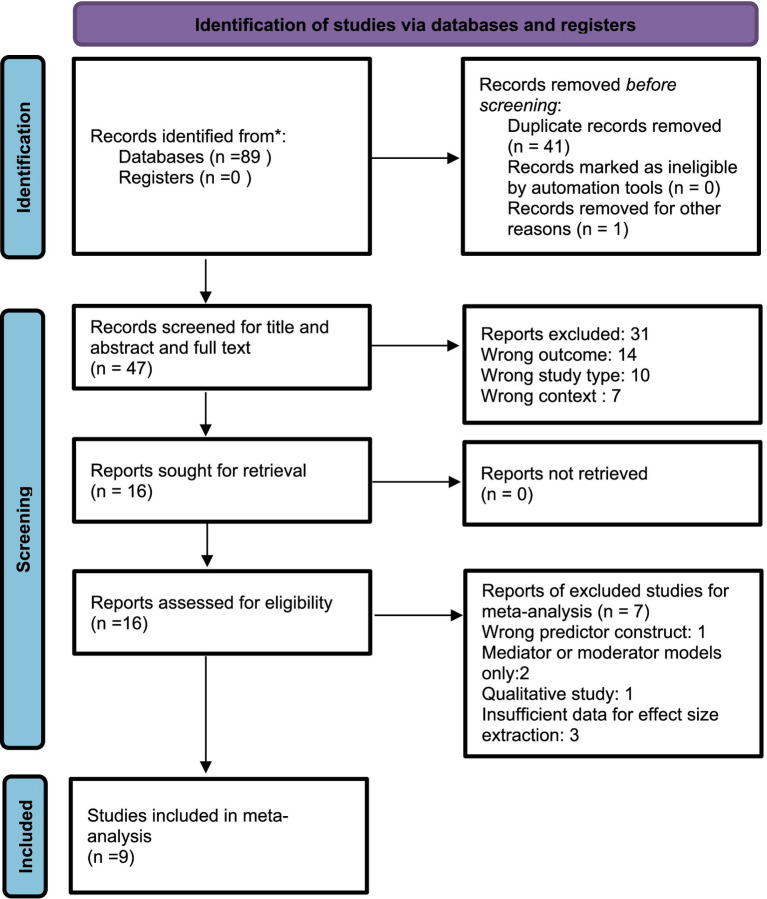
PRISMA flow diagram of study selection. Of 89 records identified, 16 met the inclusion criteria; nine were included in the meta-analysis and seven were excluded but included in narrative analysis.

The database search identified 89 records, with 16 meeting the inclusion criteria (Figure 1). Nine contributed quantitative data to the meta-analysis; seven were excluded due to insufficient data, a non-quantitative design, or a focus on mediators or moderators (Figure 1). The included studies (Table 1) were drawn from diverse regions, including Europe, North America, and Asia, and were conducted in various settings such as healthcare, corporate, and education. Sample sizes ranged from under 100 to over 1,800 participants. Eight studies were cross-sectional surveys, although the rest used longitudinal or experimental designs. Dark Triad traits were assessed using validated instruments like the SD3 and Dirty Dozen, while bullying outcomes were measured with validated indices or adapted questionnaires.

**Table 1:** Study characteristics.

Study (year)	Country/setting	Sample and role focus	Design and sampling	Risk of bias	Concise key finding
Sheng et al. (2025)	China, hospital (nursing)	292 nurses + 50 supervisors; supervisors	2-wave time-lagged; convenience	Moderate	Subordinates’ CWB predicted abusive supervision; effect buffered by sleep quality and amplified by narcissism.
Jang et al. (2025)	Korea, ICU	47 nurses	Quasi-experiment (MBAP); voluntary	Moderate	MBAP reduced narcissistic vulnerability, distortions, shame; increased mentalization; lowered bullying.
Braun et al. (2024)	Germany/UK	Supervisor samples (*N* = 320/326/292)	Survey + experiments	Moderate	Vulnerable narcissism predicted abusive supervision via internal attributions; grandiose not predictive.
Jang et al. (2023)	Korea, ICU	416 nurses	Cross-sectional survey	Moderate	Dark traits and perfectionistic self-presentation predicted bullying; narcissistic vulnerability predicted victimisation.
Feng et al. (2023)	USA and China	Managers (*n* = 355); leaders + teams (*n* = 298, *n* = 1,252 subs)	Two-wave multisource	Low–Moderate	Leader Machiavellianism predicted abusive supervision via guanxi; effects stronger with high team guanxi.
Preston et al. (2022)	USA	331 employees	Cross-sectional MTurk	Moderate	Disinhibition/Antisocial predicted CWB; Meanness → interpersonal CWB; Boldness buffered harms.
Fernandez-del-Rio et al. (2021)	Spain	613 employees	Cross-sectional survey	Moderate	Dark tetrad (esp. sadism, narcissism) predicted bullying perpetration; agreeableness protective.
Priesemuth and Bigelow (2020)	Canada/USA	111 dyads + 160 supervisors	CIT field studies; time-lagged	Moderate	Abusive supervision reduced social worth and OCB; psychopathy moderated persistence.
Dåderman and Ragnestål-Impola (2019)	Sweden	247 employees	Cross-sectional survey	Moderate	Machiavellianism and psychopathy predicted bullying; narcissism ns when controlling others.
Carre et al. (2018)	USA	481 MTurk	Cross-sectional survey	Moderate	Psychopathy (meanness, disinhibition) predicted workplace deviance and harassment proclivity.
Pilch and Turska (2015)	Poland	117 employees	Cross-sectional survey	Moderate	Machiavellianism predicted bullying perpetration; bullies scored higher; culture moderated effects.
Wang and Jiang (2014)	China	403 employees	Two-wave survey	Moderate	Abusive supervision → deviance; narcissism moderated (stronger effect for high narcissism).
Burton and Hoobler (2011)	USA	262 MBA + coworkers	Cross-sectional mediation	Moderate	Abusive supervision → aggression via injustice; narcissism intensified aggression under low justice.
Kiazad et al. (2010)	Australia and Philippines	92 dyads (AU); 200 dyads (PH)	Cross-sectional + 2-wave	Low–Moderate	Supervisor Machiavellianism → abusive supervision via authoritarian leadership; OBSE amplified in collectivist setting.
Pullen and Rhodes (2008)	UK	Qualitative case leaders	Interpretive case	High (qualitative)	Leadership identity framed as narcissistic and gendered; self-centered power practices.
Wislar et al. (2002)	USA, university	1,880 employees (drinkers)	Two-wave survey	Moderate	Harassment/abuse predicted problem drinking; personality traits partly explained differences.

Summary of study characteristics, risk-of-bias (RoB), funding, and key findings (*N* = 16). Studies are listed in descending order of publication year for clarity.

Across included studies, there was variability in how dark personality traits were operationalized. Some studies assessed all three Dark Triad traits concurrently, whereas others examined only one or two traits in isolation. A small number of studies additionally incorporated broader dark personality frameworks, such as the Dark Tetrad, typically including everyday sadism. However, such studies were limited in number and varied substantially in measurement and outcome definitions, precluding quantitative synthesis beyond the Dark Triad traits.

The included studies also differed in methodological design and cultural context. Most employed cross-sectional survey designs, whereas a smaller number used time-lagged, longitudinal, quasi-experimental, or multi-source approaches. Studies were conducted across diverse cultural settings, including North America, East Asia, and Europe, with the United States and Korea most frequently represented. These differences in design and context likely contributed to the substantial heterogeneity observed across pooled analyses.

### Risk of bias

In Table 1, 13 studies were judged to have “Moderate risk” of bias, largely due to cross-sectional design, potential selection bias, and limited adjustment for confounding. Three were rated as lower risk, particularly those with multi-wave or multi-source designs. None were free of bias in all domains. Detailed assessments are provided in Appendix Table S3.

### Meta-analysis

**Figure 2:**
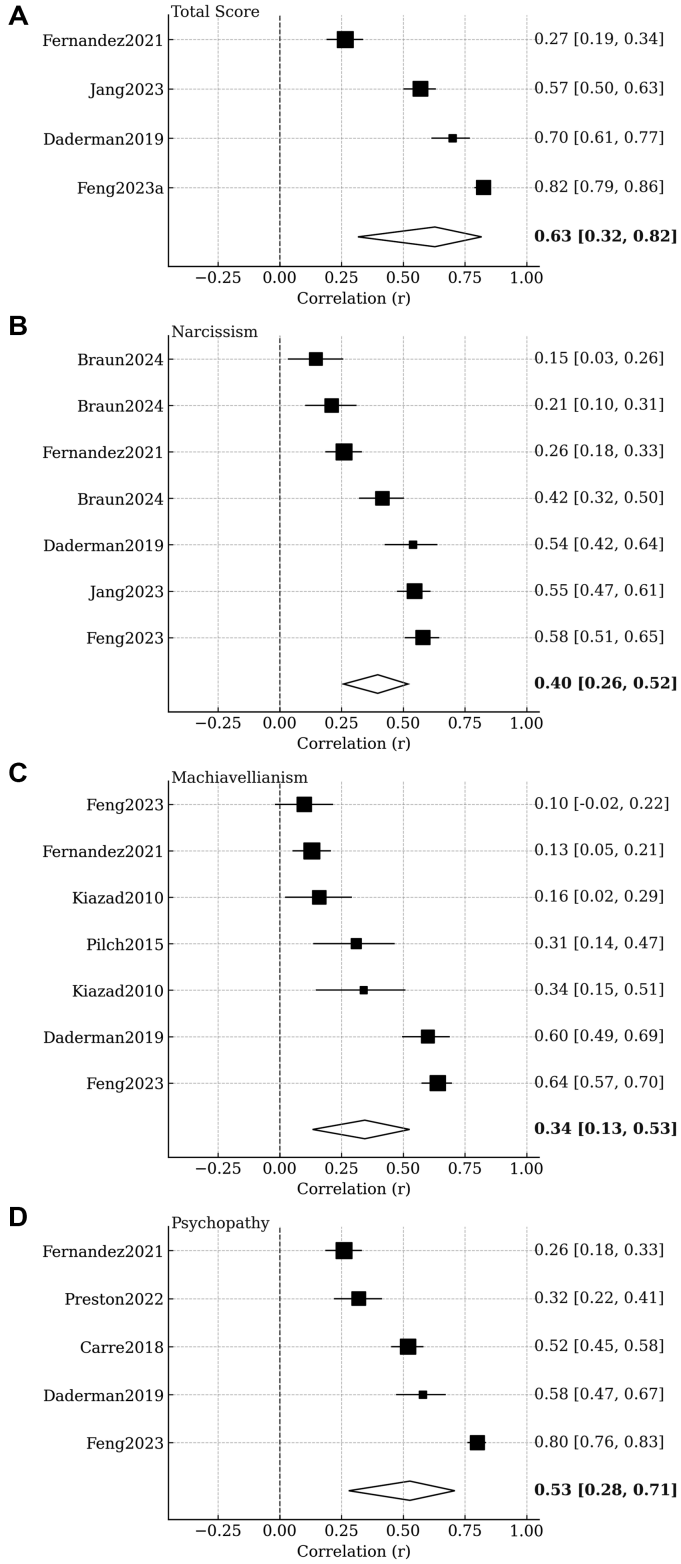
Forest plots of the association between Dark Triad traits and workplace bullying. Squares indicate individual study effects with size proportional to random-effects weight; horizontal lines show 95% confidence intervals. The diamond indicates the pooled DerSimonian-Laird random-effects estimate. Values at right are *r* with 95% CIs. Differences in the number of studies per panel reflect variation in available data across traits, as not all primary studies reported every Dark Triad dimension. **(A)** Total score; **(B)** Narcissism; **(C)** Machiavellianism; **(D)** Psychopathy.

Pooled analyses indicated that all Dark Triad personality traits were positively associated with bullying perpetration (Table 2 and Figure 2). The composite Dark Triad score showed a large, pooled correlation (*r* = 0.63 [95% CI 0.32–0.82]; *k* = 4). Psychopathy yielded the largest individual association (*r* = 0.53 [95% CI 0.28–0.71]; *k* = 5). Narcissism and Machiavellianism demonstrated moderate effects (*r* = 0.40 and *r* = 0.35 respectively). Substantial heterogeneity was observed across syntheses (*I*^2^ > 90%). Subgroup analyses showed consistently positive associations across outcome subtypes (bullying perpetration, abusive supervision, interpersonal deviance), though differences between subtypes were not statistically significant (Table 3). Prediction intervals were wide, indicating considerable between-study variation. Studies varied in the instruments used to assess Dark Triad traits, most commonly the SD3 and the Dirty Dozen. However, the number of studies per trait and per instrument was limited, restricting the feasibility and interpretability of formal moderator analyses by measurement scale. Sensitivity checks indicated that the overall direction and relative magnitude of associations were consistent across instruments, although precision was reduced in subsets defined by specific measures.

**Table 2:** Summary of syntheses (A1–A4) and sensitivity (S1).

Analysis ID	Outcome set	Trait/exposure	Model (RE method)	k	Total N†	Pooled r (95% CI)	Q (df)*	I^2^%	τ^2^ (DL)	95% prediction interval	Plot
A1 Primary	Interpersonal perpetration (bullying + abusive supervision + CWB-I)	DT total	Random-effects (DL)	4	1,556	0.628 [0.317, 0.817]	191.31 (3)	98.4	0.172	−0.171 to 0.929	
A2	Interpersonal perpetration (bullying + abusive supervision + CWB-I)	Narcissism	Random-effects (DL)	7	2,494	0.397 [0.258, 0.519]	91.03 (6)	93.4	0.041	−0.006 to 0.689	
A3	Interpersonal perpetration (bullying + abusive supervision + CWB-I)	Machiavellianism	Random-effects (DL)	7	1822	0.345 [0.133, 0.526]	130.94 (6)	95.4	0.087	−0.255 to 0.753	
A4	Interpersonal perpetration (bullying + abusive supervision + CWB-I)	Psychopathy	Random-effects (DL)	5	1952	0.527 [0.279, 0.709]	173.01 (4)	97.7	0.113	−0.137 to 0.864	
S1 (Sensitivity)	Add CWB-total/CWB-O (mixed/organizational deviance)	Specify	Random-effects (DL)	0	0	N/A (no eligible studies in current dataset)					

CWB-I, Counterproductive Work Behaviors – Individual. Analyses A1–A4 represent the four primary random-effects models (Dark Triad total, narcissism, Machiavellianism, psychopathy); S1 = sensitivity analysis including mixed CWB outcomes. Studies are ordered by descending year of publication.

**Table 3:** Outcome subtype moderators by trait.

Trait	Outcome subtype	*k*	Pooled r (95% CI)	95% PI	*Q*	*I*^2^%	τ^2^	Q_between (df, p)
Narcissism	Bullying perpetration	3	0.45 [0.23, 0.63]	−0.00 to 0.75	35.27	94.3	0.047	0.58 (1, *p* = 0.445)
Narcissism	Abusive supervision	4	0.35 [0.13, 0.54]	−0.14 to 0.70	53.87	94.4	0.053	0.58 (1, *p* = 0.445)
Machiavellianism	Bullying perpetration	3	0.36 [0.02, 0.63]	−0.31 to 0.79	42.32	95.3	0.094	0.02 (1, *p* = 0.887)
Machiavellianism	Abusive supervision	4	0.33 [−0.00, 0.60]	−0.40 to 0.80	80.77	96.3	0.122	0.02 (1, *p* = 0.887)
Psychopathy	Bullying perpetration	2	0.43 [0.07, 0.69]	−0.20 to 0.81	20.79	95.2	0.075	2.83 (2, *p* = 0.243)
Psychopathy	Abusive supervision	1	0.80 [0.76, 0.83]	0.76 to 0.83	0.00	0.0	0.000	2.83 (2, *p* = 0.243)
Psychopathy	Interpersonal deviance (CWB-I)	2	0.43 [0.21, 0.60]	0.05 to 0.70	11.65	91.4	0.027	2.83 (2, *p* = 0.243)

CWB-I, counterproductive work behavior – individual.

### Narrative synthesis

Beyond pooled estimates, clear patterns emerged across the 16 studies. Machiavellianism was a reliable predictor of bullying, particularly in competitive, market-oriented organizational cultures, while its association with perpetration weakened in more collaborative environments, suggesting strong context dependence. Supervisors scoring higher on psychopathy, as measured using the SD3 in several studies, appeared less constrained by reputational costs, allowing abusive supervision to persist. Narcissism showed more subtle effects. Vulnerable narcissism reliably predicted abusive supervision through maladaptive attributions and shame, whereas grandiose narcissism was less strongly implicated. Contextual moderators further shaped outcomes: for example, abusive supervision provoked retaliatory aggression through injustice perceptions, particularly among employees high in narcissism. Supervisors were more likely to engage in abuse when experiencing identity threat and low moral identity, and situational stressors such as job insecurity combined with leader narcissism to heighten aggression intentions. In all, these findings highlight that dark traits exert their influence within a wider matrix of organizational climate, justice perceptions, and leader-follower dynamics.

### Bias and robustness checks

**Figure 3:**
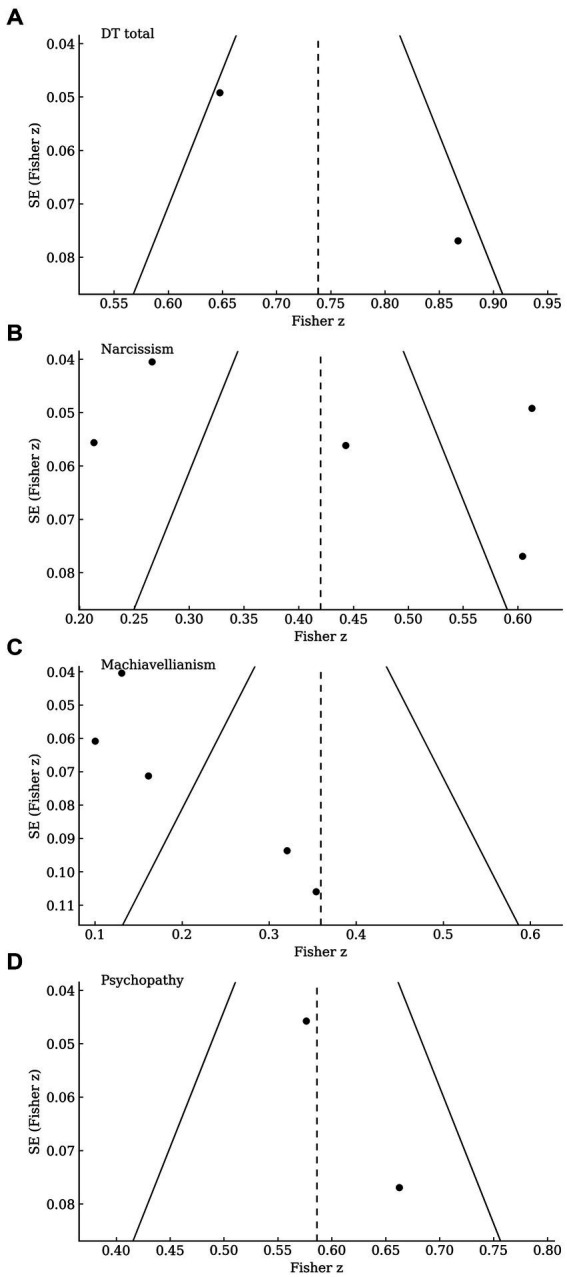
Funnel plots of small-study effects for the association between Dark Triad traits and workplace bullying. **(A)** Total score (DT-total); **(B)** Narcissism; **(C)** Machiavellianism; **(D)** Psychopathy. Each point is a study’s effect on the Fisher-z scale; the *y*-axis shows its standard error (larger studies appear lower). The vertical dashed line marks the random-effects pooled estimate; sloping lines indicate the expected 95% limits (ẑ ± 1.96 × SE). Visual asymmetry suggests potential small-study effects or publication bias; Egger’s regression intercept *p*-values were **(A)** = 0.24 (*k* = 4), **(B)** = 0.60 (*k* = 7), **(C)** = 0.61 (*k* = 7), **(D)** = 0.45 (*k* = 5), noting limited power when *k* < 10.

Funnel plots (Figure 3) suggested no systematic small-study bias, although statistical tests were underpowered due to small k. Leave-one-out analyses showed stable pooled estimates, and alternative random-effects estimators produced results consistent with DerSimonian and Laird models (DerSimonian and Laird, 1986), confirming robustness (Table 4).

**Table 4:** Bias and robustness checks (Egger + PM–KH).

Synthesis	Check	Result	Takeaway
DT total (A1)	Small-study effects (Egger)	Intercept = 20.86 (SE = 17.58), *t* = 1.19, df = 2, *p* = 0.357, *k* = 4	Under-powered (k < 10); interpret cautiously
DT total (A1)	Leave-one-out	Max Δ pooled *r* = 0.097 (dropping Feng2023a)	Large *Δ* suggests influence
DT total (A1)	Influence/outliers	Std residuals >2: None	Inspect if listed
DT total (A1)	Spec check (FE vs. RE)	FE *r* = 0.567 vs. RE *r* = 0.628	If materially different, heterogeneity matters
Narcissism	Small-study effects (Egger)	Intercept = 4.75 (SE = 9.01), *t* = 0.53, df = 5, *p* = 0.620, *k* = 7	Under-powered (k < 10); interpret cautiously
Narcissism	Leave-one-out	Max Δ pooled *r* = 0.037 (dropping Braun2024)	Stable
Narcissism	Influence/outliers	Std residuals >2: None	Inspect if listed
Narcissism	Spec check (FE vs. RE)	FE *r* = 0.385 vs. RE *r* = 0.397	If materially different, heterogeneity matters
Machiavellianism	Small-study effects (Egger)	Intercept = 3.19 (SE = 6.24), *t* = 0.51, df = 5, *p* = 0.632, *k* = 7	Under-powered (*k* < 10); interpret cautiously
Machiavellianism	Leave-one-out	Max Δ pooled *r* = 0.065 (dropping Feng2023)	Large Δ suggests influence
Machiavellianism	Influence/outliers	Std residuals >2: None	Inspect if listed
Machiavellianism	Spec check (FE vs. RE)	FE *r* = 0.317 vs. RE *r* = 0.345	If materially different, heterogeneity matters
Psychopathy	Small-study effects (Egger)	Intercept = 11.88 (SE = 15.58), *t* = 0.76, df = 3, *p* = 0.501, *k* = 5	Under-powered (k < 10); interpret cautiously
Psychopathy	Leave-one-out	Max Δ pooled *r* = 0.102 (dropping Feng2023)	Large Δ suggests influence
Psychopathy	Influence/outliers	Std residuals >2: None	Inspect if listed
Psychopathy	Spec check (FE vs. RE)	FE *r* = 0.493 vs. RE *r* = 0.527	If materially different, heterogeneity matters
DT total (A1)	Model sensitivity (PM–KH)	*r* = 0.628 [0.132, 0.872] (τ^2^_PM = 0.141, *H*^2^ = 1.00, t_crit = 3.18)	Similar to DL → conclusions stable
Narcissism	Model sensitivity (PM–KH)	*r* = 0.397 [0.220, 0.549] (τ^2^_PM = 0.042, *H*^2^ = 1.00, t_crit = 2.45)	Similar to DL → conclusions stable
Machiavellianism	Model sensitivity (PM–KH)	*r* = 0.344 [0.108, 0.544] (τ^2^_PM = 0.068, *H*^2^ = 1.00, t_crit = 2.45)	Similar to DL → conclusions stable
Psychopathy	Model sensitivity (PM–KH)	*r* = 0.527 [0.174, 0.760] (τ^2^_PM = 0.106, *H*^2^ = 1.00, t_crit = 2.78)	Similar to DL → conclusions stable

Dark Triad (DT; Machiavellianism, narcissism, psychopathy); Dark Triad total composite (DT-total); Analysis 1 worksheet label (A1); Egger’s regression test for small-study effects (Egger); standard error (SE); t-statistic (t); degrees of freedom (df); p-value (p); number of studies/effects (k); change/difference (Δ); fixed-effects model (FE); random-effects model (RE); standardized (studentized) residuals (Std residuals); Pearson correlation coefficient (r); Paule–Mandel τ^2^ estimator with Knapp–Hartung adjustment (PM–KH); between-study variance (τ^2^); Paule–Mandel estimate of τ^2^ (τ^2^_PM); heterogeneity index (H^2^); critical value from the t-distribution (t_crit); DerSimonian–Laird τ^2^ estimator (DL); confidence interval (CI).

## Discussion

This systematic review and meta-analysis suggested that the socially aversive Dark Triad personality traits, are meaningfully associated with workplace bullying perpetration. Across models and sensitivity checks, psychopathy emerged as the strongest and most consistent correlate, with Machiavellianism and narcissism showing moderate associations. Across models and sensitivity checks, psychopathy emerged as the strongest and most consistent correlate, with Machiavellianism and narcissism showing moderate associations. The rank order (psychopathy > narcissism ≈ Machiavellianism) aligns with theory: callous affect and reduced affective empathy – rather than a total lack of empathic capacity – facilitate instrumental aggression (Ritchie et al., 2022; Wislar et al., 2002); impulsivity and thrill seeking can lower thresholds for norm violations (Pink et al., 2022); and strategic manipulation supports goal-directed mistreatment when accountability is weak (Cai et al., 2023; De Hoogh et al., 2021). These findings narrow an evidence gap created by prior syntheses that mixed perpetration and victimization or collapsed interpersonal aggression with organizational deviance (Bowling and Beehr, 2006; Horsten et al., 2023; O'Boyle et al., 2012; Özer and Escartín, 2023). For example, A recent systematic review by Özer and Escartín (2023) catalogued multilevel antecedents, moderators, mediators, and outcomes of bullying perpetration across studies; however, it did not quantitatively pool personality–perpetration associations (e.g., Dark Triad) nor differentiate effect sizes by outcome subtype, leaving the strength and specificity of these links unresolved. By focusing explicitly on perpetration, we quantify the personality-aggression link that organizations must manage.

From a theoretical perspective, the findings support a person–context interaction account of workplace bullying perpetration. Dark personality traits appear to function as dispositional risk factors whose expression depends on organizational opportunity structures, such as power asymmetries, weak accountability, and permissive climates. Traits characterized by callousness, manipulation, and entitlement may increase the likelihood of bullying when situational constraints are low, rather than operating as deterministic causes of mistreatment. This interpretation aligns with contemporary models of workplace aggression that emphasize the joint influence of individual dispositions and contextual affordances and cautions against trait-based explanations that neglect organizational responsibility (Schat and Frone, 2011).

Framed as an occupational and public health issue, the results reinforce that bullying is associated with substantial individual and societal harms. Mental health effects include anxiety, depressive symptoms, burnout, and features of post-traumatic stress (Ashraf et al., 2024). Physical effects include sleep disruption (Niedhammer et al., 2009; Nielsen et al., 2020), musculoskeletal pain (Vignoli et al., 2015), and cardiometabolic risk (Kivimäki et al., 2003). Conceptually plausible mechanisms connect these outcomes to bullying as a chronic social stressor, including activation of the hypothalamic–pituitary–adrenal axis and sympathetic system, glucocorticoid dysregulation, and low-grade systemic inflammation, which translate psychosocial strain into disease risk (Rivara et al., 2016). From a systems perspective, these harms expand beyond the individual to organizational costs (turnover intentions, absenteeism/presenteeism, productivity loss) (Brouwer et al., 2023) and societal burden (healthcare utilization, compensation claims) (Isham et al., 2021).

At the same time, the evidence cautions against trait determinism and emphasizes the primacy of context. The work environment hypothesis suggests bullying proliferates in climates marked by destructive or laissez-faire leadership (Nielsen et al., 2024), low organizational justice (Abou Hashish et al., 2024), role ambiguity (Blomberg et al., 2024), poor job design (high demands/low resources) (Bjaalid et al., 2022), and power asymmetries (Soylu and Sheehy-Skeffington, 2015). A strong Psychosocial Safety Climate (PSC), the shared perception that management prioritizes psychological health, functions as a “cause of the causes,” dampening the opportunity structure for aggression (Fattori et al., 2022). Our synthesis supports a multilevel account in which (i) individual dispositions (e.g., psychopathy’s callousness, Machiavellianism’s strategic manipulation, and specific narcissism facets) interact with (ii) organizational culture (PSC, justice, ethical climate) and (iii) situational stressors (job insecurity, resource scarcity) to enable or constrain bullying. Of note, effects for narcissism were more variable across studies, consistent with theoretical distinctions between grandiose and vulnerable narcissism; the latter may be more reactive to ego threat, whereas the former is expressed where power and impunity are available (Aslinger et al., 2022).

### Robustness, heterogeneity, and sensitivity

We observed heterogeneity across traits and outcome subtypes (bullying perpetration, abusive supervision, CWB-I). This likely reflects measurement differences, sampling frames (occupational sectors and countries), and model adjustments. Small-study bias diagnostics (Egger tests) were under-powered and therefore interpreted cautiously. Leave-one-out analyses indicated influence for a small number of effects in the psychopathy and Machiavellianism sets, while narcissism appeared stable under case deletion. Fixed-versus random-effects comparisons suggested that heterogeneity matters, and model sensitivity using Paule-Mandel with Knapp-Hartung adjustments produced conclusions consistent with the primary models. Therefore, these checks increase confidence that the direction and relative magnitudes of the pooled associations are not artefacts of a single estimator or idiosyncratic study.

Differences in study design and cultural context provide important insight into the observed heterogeneity. Cross-sectional designs relying on single-source self-report data may inflate associations through shared-method variance, whereas longitudinal, experimental, or multi-source designs offer stronger temporal and causal inference and may yield more conservative estimates. Cultural context may further shape how dark personality traits are expressed and perceived in workplace interactions, particularly in relation to power distance, authority norms, and reporting practices. Consequently, effect sizes should be interpreted as average tendencies across heterogeneous contexts rather than as uniform estimates applicable to all organizational or cultural settings.

### Practical implications

The translation of these findings is organizational, not clinical. Although personality traits should not be used as exclusionary screening tools, awareness of dark personality tendencies may inform leadership development, governance, and prevention strategies. Organizational safeguards, such as clear behavioral standards, robust reporting mechanisms, procedural justice, and a strong psychosocial safety climate, may limit opportunities for dispositional risk to translate into sustained bullying. Interventions that focus on accountability, ethical leadership, and work design may therefore be more effective than approaches that target individual traits in isolation.

Personality assessments should not be used as blunt screening or exclusion tools in recruitment or promotion, but rather as one component of a broader evaluation strategy. Importantly, the persistence of psychopathic leadership behaviors may reflect structural, rather than individual, determinants. Positions that reward dominance and power signaling inherently attract individuals high in callous-manipulative traits. By contrast, rotational or collective leadership models, such as those adopted in some UK universities, minimize such selection pressures by decoupling power from positional permanence. In this view, psychopathy is selected, not created, by systems that conflate authority with personal dominance. Reforms that flatten hierarchies or distribute decision-making power may therefore constitute one of the most effective safeguards against toxic supervision. We recommend integrating these measures with contextual judgement, behavioral feedback, and leadership-development frameworks to mitigate misuse while harnessing their diagnostic value. In addition to, a risk-management approach consistent with occupational health and safety principles is warranted: build personality awareness and behavioral accountability into supervision training, using 360-degree feedback and mentoring to surface blind spots, especially in leaders with grandiose or callous tendencies. Establish system safeguards through zero-tolerance policies, confidential and credible reporting channels, timely investigation protocols, and just-culture responses that separate accountability from blame. Improve work design to reduce opportunities for bullying by clarifying roles, allocating workloads fairly, ensuring transparent decision-making, and embedding procedural justice in performance management. Strengthen the PSC via visible senior-management commitment to psychological health, dedicated resources for prevention, and routine monitoring of interpersonal climate. Additionally, provide support and remediation by offering coaching for perpetrators who can change and ensuring access to evidence-based assistance for targets and teams (e.g., restorative practices, conflict management).

### Strengths and limitations

Strengths include prospective preregistration, a comprehensive multi-database search, duplicate screening and extraction, and transparent risk-of-bias procedures adapted from the JBI checklist. We synthesized trait-specific and DT-total effects and examined outcome subtypes to avoid conflating interpersonal aggression with broader counterproductive behaviors.

Limitations warrant restraint in interpretation. The included studies sampled participants from diverse occupational contexts, including healthcare, corporate, education, and mixed-sector workforces. While this diversity reflects the widespread nature of workplace bullying, it limits the generalizability of pooled estimates to specific occupational settings and likely contributes to between-study variability.

Methodological heterogeneity also arose from differences in the operationalization of dark personality traits. Some studies assessed individual Dark Triad traits in isolation, whereas others examined partial combinations or the full triad, which may influence observed effect sizes and comparability across studies. Although broader frameworks such as the Dark Tetrad are theoretically relevant, the limited number and heterogeneity of such studies currently restrict the feasibility of quantitative synthesis or moderator analyses beyond the Dark Triad.

In addition, most included studies were cross-sectional and relied on single-source self-report data, which may inflate associations through shared-method variance and preclude causal inference. The modest number of meta-eligible effects per trait further constrained precision and limited the power of small-study bias diagnostics. Finally, although our focus on the Dark Triad ensured theoretical coherence and methodological comparability, related constructs such as everyday sadism and protective traits (e.g., empathy or honesty–humility) were outside the scope of this review and warrant future synthesis.

Measurement-related limitations should also be noted. Brief instruments such as the Dirty Dozen are widely used in organizational research due to their efficiency, yet their psychometric properties and construct coverage have been questioned. Variation in scale length and dimensional coverage may have contributed to between-study variability in effect sizes. Accordingly, pooled estimates should be interpreted as reflecting broad trait tendencies rather than fine-grained facet-level associations. Future research would benefit from greater consistency in measurement and from using instruments with stronger construct validity when examining dark personality traits in workplace contexts.

### Future directions

To refine estimates and inform prevention, future research should prioritize longitudinal, multi-wave, and multi-source designs to establish temporal order and reduce shared-method bias; report meta-analytic inputs transparently (e.g., r, n, and confidence intervals) and make materials openly available. Trait delineation should also improve, distinguishing fine-grained facets such as grandiose versus vulnerable narcissism; psychopathy’s triarchic dimensions of boldness, meanness, and disinhibition, which map onto primary (bold–mean) and secondary (disinhibited) psychopathy profiles (Patrick et al., 2009; Sellbom and Drislane, 2021); and tactical versus cynical forms of Machiavellianism. The scope should further expand to the Dark Tetrad and to protective dispositions such as empathy, honesty-humility, mentalization, and resilience. Future studies should pre-register and test contextual moderators that determine when traits translate into harm (e.g., psychosocial safety climate, organizational justice, leadership style, power asymmetries, job insecurity, and role ambiguity). Finally, organizational interventions should be evaluated using field or quasi-experimental designs, such as staggered PSC initiatives, leadership feedback and coaching, or changes to accountability and reporting systems, with follow-ups tracking downstream health and productivity outcomes. Importantly, this review does not imply that dark personality traits inevitably lead to bullying, but rather that they represent one component within a broader multilevel system of risk.

## Conclusion

Machiavellianism, narcissism, and psychopathy are consistent and meaningful risk factors for workplace bullying perpetration, with psychopathy showing the strongest association. By isolating perpetration and integrating sensitivity analyses, this review clarifies how aversive dispositions translate into harmful conduct when organizational conditions permit. Effective prevention requires a multilevel strategy: leadership development and governance that reward respectful behavior, robust system safeguards and fair processes, high psychosocial safety climate, and work design that limits opportunities for interpersonal harm. Workplace bullying is both an organizational hazard and a public health priority; coordinated action across psychology, management, and occupational health is warranted.

## Data Availability

The original contributions presented in the study are included in the article/Supplementary material, further inquiries can be directed to the corresponding author/s.
