# Supplementary material for: Dark Triad traits and workplace bullying: a systematic review and meta-analysis of personality, power, and psychosocial safety
Source: Front Psychol. 2026 Mar 4;17:1738277. doi: 10.3389/fpsyg.2026.1738277 (PMC12995606; doi:10.3389/fpsyg.2026.1738277)
Supplement: Supplementary file 1 [file Data_Sheet_1.pdf]

Appendix 1. Full search protocol and data-extraction materials

A. Registration and reporting

This protocol is prospectively registered on the Open Science Framework (OSF: 10.17605/OSF.IO/TVFKC). The review will be reported in accordance with PRISMA 2020.

B. Eligibility (PICOS-E)

Population. Employed adults (≥18 years) in workplace settings; student-only/school bullying samples excluded unless participants are employed.

Exposures. Personality traits with a focus on the Dark Triad (Machiavellianism, narcissism, psychopathy) and validated trait models; constructs not analysed as personality are excluded.

Comparator. Not applicable (associational designs).

Outcomes. Workplace bullying/mobbing perpetration (also abusive supervision; interpersonal deviance); validated scales (e.g., NAQ/NAQ-R, LIPT) or self-label approaches.

Study design. Observational (cross-sectional, cohort, case-control); qualitative-only, case studies, editorials, and reviews excluded.

Setting. Any sector and country/region.

Language/time. No language limit; databases from inception to search date.

C. Information sources and search strategy

Databases: PubMed, PsycINFO, Embase (Ovid), Scopus, Web of Science. Hand-searching of reference lists and forward citations of included studies. Optional grey literature: ProQuest Dissertations, OpenGrey.

Core concept blocks: Workplace (workplace/work environment/occupation/employee/organisation), AND bullying (bully/mobbing/negative acts/harassment/mistreat*/abusive supervision/toxic leadership), AND Dark Triad terms (Machiavell*/narciss*/psychopathy/“dark triad”/SD3/Dirty Dozen/NPI/Mach-IV/LSRP), AND perpetration/aggression/harassment.

Example strings (verbatim):

PubMed: (("workplace"[Title/Abstract] OR "work environment"[Title/Abstract] OR occupation*[Title/Abstract] OR employee*[Title/Abstract] OR organization*[Title/Abstract] OR organisation*[Title/Abstract]) AND (bully*[Title/Abstract] OR mobbing[Title/Abstract] OR "negative acts"[Title/Abstract]

OR harassment[Title/Abstract] OR mistreat*[Title/Abstract]) AND (Machiavell*[Title/Abstract] OR narciss*[Title/Abstract] OR psychopathy[Title/Abstract] OR "dark triad"[Title/Abstract]) AND (perpetrat*[Title/Abstract] OR aggress*[Title/Abstract] OR harass*[Title/Abstract])) AND English[lang]

PsycINFO (fielded): ((workplace or (work adj3 (place or environment)) or occupation* or employee* or organi?ation*).ti,ab. and (bully* or mobbing or "negative act*" or harass* or mistreat* or incivil* or "abusive supervision" or "toxic leadership").ti,ab. and (Machiavell* or narciss* or psychopathy or "dark triad" or "Short Dark Triad" or "Dirty Dozen" or "Narcissistic Personality Inventory" or "Mach-IV" or LSRP).ti,ab. and (perpetrat* or aggress* or harass*).ti,ab.) not ((student* or school or adolescen* or child*).ti,ab.)

Search management: Export all records with full metadata; de-duplicate in EndNote/Zotero and import to Rayyan for screening; document search dates and yields for PRISMA flow.

D. Screening and study selection

Two reviewers (SS, LY) independently screen titles/abstracts, then full texts, recording reasons for exclusion. Discrepancies resolved by discussion or third reviewer. Agreement (κ) will be reported. PRISMA 2020 flow diagram will document identification, screening, eligibility, and inclusion.

E. Data-extraction instruments (templates)

We will use the following standardised forms (excerpted headers shown here; full column sets in the working spreadsheets).

E1. Study characteristics form (Table A1)

Study (Author, Year)	Country/Region	Sector/Setting	Sample (n) & demographics	Role focus	Design & time-lag	Sampling & response rate	Funding/ COI	Concise finding

E2. Detailed exposure/outcome/analysis extraction (Table A2)

Trait measure (instrument; α)	Outcome (instrument; α; chronicity)	Other variables	Role/ target clarity	Design specifics	Model/level /covariates	Effect size (type, value, 95	Adjusted vs unadjusted	Common-method risk	Context moderators	Replicability notes

						% CI)				

E3. Quality appraisal checklist (Table A3)

Tool	Clear aims	Appropriate methodology	Recruitment	Exposure validity	Outcome validity	Confounding	Statistical methods	Missing data	Ethics	Clarity of findings	External validity	Overall judgement

F. Risk of bias and certainty of evidence

Risk of bias will be assessed independently by two reviewers using an adapted CASP tool for observational studies, with domain-level judgements and an overall rating (Low/Some concerns/High). Certainty of evidence will be graded using the Grading of Recommendations, Assessment, Development and Evaluations (GRADE) approach.

G. Effect size specification and computation

The primary effect is the correlation (r) between each Dark Triad trait and bullying perpetration. We will: (i) prefer validated perpetration measures; (ii) transform effects to Fisher's z for meta-analysis and back-transform to r; (iii) convert OR, d, t, and standardised β to r when defensible; and (iv) clearly flag adjusted vs unadjusted estimates.

H. Synthesis and statistical analysis

Primary models use random-effects (REML) on Fisher's z. Dependence among multiple effects will be addressed via three-level meta-analysis or robust variance estimation. Heterogeneity will be summarised with τ^2, I^2, Q and 95% prediction intervals. Pre-specified moderators include role, trait family/trait-specific effects, instruments, sector/region, design, sample, year, and adjustment status. Influence diagnostics (leave-one-out; studentised residuals; Cook's distance) will be reported.

I. Small-study effects and publication bias

Funnel plots (Fisher's z) and Egger's regression (or meta-regression with SE) will be performed, acknowledging limited power when $k<10$. Sensitivity analyses may include trim-and-fill and selection models.

J. Handling special cases

We will address overlapping samples, multiple trait/outcome effects per study (retained with dependence modelling), dichotomous vs continuous outcomes (analysed separately or converted where defensible), and translate non-English articles as needed for extraction.

K. Data management and transparency

All search logs, extraction forms, analytic code (R metafor/clubSandwich) and the final dataset will be shared via the OSF project linked to the registration DOI. Author contact will be attempted for missing or unclear data (two emails spaced ≥10 days).

L. Amendments

Any deviations from this protocol (e.g., changes to eligibility, outcomes, or analyses) will be dated, described, and justified in the OSF record and reported in the manuscript's Methods.
