# Supplementary material for: Dark Triad traits and workplace bullying: a systematic review and meta-analysis of personality, power, and psychosocial safety
Source: Front Psychol. 2026 Mar 4;17:1738277. doi: 10.3389/fpsyg.2026.1738277 (PMC12995606; doi:10.3389/fpsyg.2026.1738277)
Supplement: Supplementary file 2 [file Data_Sheet_2.pdf]

Appendix Table S1. Full-text articles excluded, with reasons

Note: Reasons categorised as Wrong outcome (n = 14), Wrong study type (n = 10), Wrong context (n = 7), consistent with the PRISMA flow.

Study (first author)	Year	Reason category	Specific reason at full-text
Srivastava et al.	2024	Wrong outcome	No bullying perpetration measured
Pryor et al.	2024	Wrong outcome	No bullying perpetration measured
O'Reilly III et al.	2024	Wrong outcome	No bullying perpetration measured
Longpré & Turner	2024	Wrong outcome	No bullying perpetration measured
Piasecki	2023	Wrong outcome	No bullying perpetration measured
de Bruin & Finkelstein	2023	Wrong outcome	No bullying perpetration measured
Collier	2023	Wrong outcome	No bullying perpetration measured
Lata & Chaudhary	2022	Wrong outcome	No bullying perpetration measured
Fritzon et al.	2017	Wrong outcome	No bullying perpetration measured
Abrams	2017	Wrong outcome	No bullying perpetration measured
Linton & Power	2013	Wrong outcome	No bullying perpetration measured
Gammon et al.	2011	Wrong outcome	No bullying perpetration measured
Song	2021	Wrong outcome	No enacted bullying perpetration (harassment intention only)
Richman et al.	1996	Wrong outcome	No bullying perpetration measured
Looi et al.	2024	Wrong study type	Review/editorial
Cohen	2024	Wrong study type	Not empirical (book/monograph)
Boddy & Taplin	2024	Wrong study type	Book
Cao et al.	2023	Wrong study type	Meta-analysis
Pineda et al.	2022	Wrong study type	Victimisation only (not perpetration)
Jaworski	2022	Wrong study type	Book
Mathieu	2021	Wrong study type	Book
Liu et al.	2021	Wrong study type	Book chapter

Blackwood & Jenkins	2021	Wrong study type	Book chapter
Myers	2019	Wrong study type	PhD thesis
Mota-Rojas et al.	2022	Wrong context	Not workplace/organisational bullying
McCarthy	2015	Wrong context	Not workplace/organisational bullying
Mizuno	2013	Wrong context	Not workplace/organisational bullying
Sharratt & Brannigan	2009	Wrong context	Not workplace/organisational bullying
Ono & Pumariega	2008	Wrong context	Not workplace/organisational bullying
Baumeister et al.	2003	Wrong context	Not workplace/organisational bullying
Epstein	1981	Wrong context	Not workplace/organisational bullying
