# Supplementary material for: Dark Triad traits and workplace bullying: a systematic review and meta-analysis of personality, power, and psychosocial safety
Source: Front Psychol. 2026 Mar 4;17:1738277. doi: 10.3389/fpsyg.2026.1738277 (PMC12995606; doi:10.3389/fpsyg.2026.1738277)
Supplement: Supplementary file 3 [file Data_Sheet_3.pdf]

Table S2 Study Characteristics -full data								
**Study (Author, Year)**	**Country / Region**	**Sector / Setting**	**Sample (n, population, occupation)**	**Role focus (Perpetrator)**	**Study Design**	**Sampling method & Response rate**	**Funding / COI**	**Key finding**
Sheng et al., 2025.*	China	Hospital setting (nursing)	292 subordinate nurses (93% female, mean age 27.3, avg. tenure 3.6 yrs); 50 nurse supervisors (76% female, mean age 38.5, avg. supervisory tenure 1.3 yrs)	Supervisors	Time-lagged survey, 2 waves, 1-month interval	Convenience sampling within a single hospital; response rate not explicitly reported	Funding not stated; no conflicts declared	Results showed that subordinates' CWB at Time 1 was positively related to their supervisors' abusive supervision at Time 2. Supervisors' sleep quality was found to mitigate the relationship between subordinates' CWB and supervisors' abusive supervision; however, the moderating effect of supervisors' sleep quantity was not significant. Moreover, supervisors' narcissism exacerbated the relationship

Table S2 Study Characteristics -full data								
**Study (Author, Year)**	**Country / Region**	**Sector / Setting**	**Sample (n, population, occupation)**	**Role focus (Perpetrator)**	**Study Design**	**Sampling method & Response rate**	**Funding / COI**	**Key finding**
								between subordinates' CWB and supervisors' abusive supervision. This study contributes to the abusive supervision literature by shifting away from the victim precipitation paradigm and placing supervisors, those in positions of power, at the forefront. Findings from the study provide insights into the design and implementation of supervisor training aimed at mitigating abusive behaviors in the workplace. © 2024 Elsevier

Table S2 Study Characteristics -full data								
**Study (Author, Year)**	**Country / Region**	**Sector / Setting**	**Sample (n, population, occupation)**	**Role focus (Perpetrator)**	**Study Design**	**Sampling method & Response rate**	**Funding / COI**	**Key finding**
								B.V., All rights reserved.
Jang, Kim & Lee, 2025*.	Republic of Korea	(Tertiary hospital ICU)	N=47 (Intervention=21; Control=26). Mean age ≈30.0 years (INT 30.14±4.51; CTRL 29.96±3.77). **Gender:** INT 0% male / 100% female; CTRL 23.1% male / 76.9% female (Total 12.8% male). **Education:** ~94% Bachelor's. **Tenure:** Total working years **6.28±4.01**; current unit **3.98±2.79**.	Nurses (bullying behaviours self-reported). **Peer-to-peer** bullying among ICU nurses (victim & perpetrator aspects).	**Quasi-experimental**, non-equivalent control group, pretest–2 weeks–4 weeks; mentalization-based antibullying program (MBAP). **Analysis:** GEE with covariates (e.g., gender).	**Voluntary recruitment** via notices/groupware; stratified by ≥/<3-years' experience; not randomized. **Dropouts:** 5/26 in intervention during assignment; final analysis N=47. **Response rate:** NR.	Funding NR; ethics approval obtained	Results: Intervention group participants showed a significant reduction in the narcissistic vulnerability subscale of pathological narcissism compared to the control group. They also exhibited a significant decrease in interpersonal cognitive distortions and an increase in mentalization scores. Regarding self-conscious emotions, the shame score significantly decreased, while

Table S2 Study Characteristics -full data								
**Study (Author, Year)**	**Country / Region**	**Sector / Setting**	**Sample (n, population, occupation)**	**Role focus (Perpetrator)**	**Study Design**	**Sampling method & Response rate**	**Funding / COI**	**Key finding**
								the guilt score significantly increased. Conclusions: MBAP can be an effective intervention for addressing workplace bullying. By targeting psychological and cognitive factors, including narcissistic vulnerability, interpersonal cognitive distortions, and self-conscious emotions, MBAP holds promise for promoting healthier interpersonal dynamics and emotional regulation among ICU nurses. Implications for

Table S2 Study Characteristics -full data								
**Study (Author, Year)**	**Country / Region**	**Sector / Setting**	**Sample (n, population, occupation)**	**Role focus (Perpetrator)**	**Study Design**	**Sampling method & Response rate**	**Funding / COI**	**Key finding**
								Nursing Management: The MBAP can be implemented broadly to prevent workplace bullying and promote a positive organizational culture in ICU settings. Future research should refine and adapt the program to accommodate the unique work characteristics and interpersonal dynamics of nurses in various settings. Workplace bullying is a deeply entrenched negative organizational issue in nursing,

Table S2 Study Characteristics -full data								
**Study (Author, Year)**	**Country / Region**	**Sector / Setting**	**Sample (n, population, occupation)**	**Role focus (Perpetrator)**	**Study Design**	**Sampling method & Response rate**	**Funding / COI**	**Key finding**
								and this program could be a highly effective intervention across diverse healthcare environments.
Braun et al., 2024	Germany (study 1&2) and UK (study 3)	Business / organisational context	Study 1: N=320 supervisors (team leaders, dept managers, business area managers, top managers); Study 2: N=326 supervisors; Study 3: N=292 supervisors	**Supervisors** (perpetrators of abusive supervision toward subordinates)	Study 1: Correlational survey. Study 2: Experimental scenario (manipulation -of-mediator). Study 3: Experimental event-recall design (manipulation -of-mediator).	Recruitment details limited; voluntary participation. Response rate **NR**.	**No conflicts of interest** reported. Funding not specified. Ethics approval reported (Durham University).	Across 3 studies, vulnerable narcissism predicted abusive supervision (self-rated/intentions). Internal attribution of failure mediated this link. Study 2 provided partial evidence for shame as serial mediator, but not replicated in Study 3. Grandiose narcissism did not show comparable effects.

Table S2 Study Characteristics -full data								
**Study (Author, Year)**	**Country / Region**	**Sector / Setting**	**Sample (n, population, occupation)**	**Role focus (Perpetrator)**	**Study Design**	**Sampling method & Response rate**	**Funding / COI**	**Key finding**
Jang, Kim & Lee (2023)	Republic of Korea	Intensive Care Units, tertiary hospitals	n = 416 ICU nurses; 91.3% female; mean age = 30.8 years (SD = 5.5); mean experience = 5.9 years (SD = 4.5)	Both victim and perpetrator roles examined	Cross-sectional, online survey	Convenience sampling via online nurse communities and hospital groupware; 432 responses, 416 valid (final response rate ~96% after exclusions)	Funded by National Research Foundation of Korea, Ministry of Education (grants 2021R1F1A1046718 and 2022R1F1A1063441). Authors declare no conflicts of interest.	Dark personality traits (esp. SD3) and perfectionistic self-presentation predicted bullying perpetration; narcissistic vulnerability predicted victimization. Mentalization negatively associated with both victimization and perpetration
Feng et al., 2023	USA and China	Study 1: Online panel (Amazon MTurk) across industries. Study 2: Large Chinese state-owned power company; 13 municipal branches; intact work teams.	Study 1: *n*=355 full-time managers (supervising ≥2 employees), two-wave survey (T1→T2). Study 2: *T1:* 1,347 subordinates (99.6%) & 298 leaders (99.3%); *T2:* 1,252	Leaders/supervisors as potential perpetrators of abusive supervision (leader-reported in Study 1; subordinate-reported and aggregated to team level in Study 2).	Study 1: Two-wave, time-lagged, single-source survey. Study 2: Two-wave, multisource, multilevel field study (leaders & subordinates).	Study 1: MTurk screening (≥97% approval, ≥1,000 prior HITs); T1 = 570; T2 = 375 (≈68% of eligible); final *n* = 355 after checks. Study 2: On-site census of units; very high response at both waves (see counts).	Funding: National Natural Science Foundation of China (NSFC) #72102225, #71572076. COI: not stated in article.	Across studies, leader Machiavellianism predicts higher abusive supervision via eader–direct-supervisor guanxi(instrumental relationship building). The indirect effect is

Table S2 Study Characteristics -full data								
**Study (Author, Year)**	**Country / Region**	**Sector / Setting**	**Sample (n, population, occupation)**	**Role focus (Perpetrator)**	**Study Design**	**Sampling method & Response rate**	**Funding / COI**	**Key finding**
			subordinates (92.9%) & 273 leaders (91.6%); multilevel (subordinates nested within leaders/teams).					stronger when team-member guanxi (TMG) is high. Abusive supervision, in turn, is associated with lower team voice and lower OCBI.
Preston et al 2021	USA	Community employees recruited online	n = 331 (after exclusions); employed adults, 51.1% female, mean age = 37 (range 19–79), diverse occupations and sectors	General employees as potential perpetrators of CWB (counterproductive workplace behaviours)	Cross-sectional survey	Recruited via Amazon MTurk; eligibility = ≥100 prior tasks with ≥95% approval; final n after exclusions 331 (response rate not reported but screened for attention checks and validity)	No funding; authors declare no conflicts of interest	Psychopathic traits showed differential associations: impulsive–antisocial traits (Disinhibition, Antisocial) strongly predicted interpersonal and organizational CWB; Boldness positively predicted OCB and buffered some maladaptive effects; Meanness predicted interpersonal CWB; interactions

Table S2 Study Characteristics -full data								
**Study (Author, Year)**	**Country / Region**	**Sector / Setting**	**Sample (n, population, occupation)**	**Role focus (Perpetrator)**	**Study Design**	**Sampling method & Response rate**	**Funding / COI**	**Key finding**
								among traits influenced outcomes.
Fernández -del-Río, Ramos-Villagrasa , & Escartín (2021)	Spain	Various organizations across sectors	n = 613 employees (54% female; mean age = 38.8 years, SD = 14.1; average job tenure = 8.4 years, SD = 10.1)	Both perpetrator and target roles measured (self-reports)	Cross-sectional survey (paper-and-pencil questionnaire)	Non-probability sampling via university students distributing surveys in workplaces; 720 distributed, 625 returned (86.8% response), final n = 613 (85.1% usable)	Supported by Gobierno de Aragón (Group S31_20D), co-funded by FEDER 2014–2020; no conflicts of interest declared	Dark Tetrad traits (especially sadism and narcissism) positively predicted bullying perpetration, while agreeableness (Big Five) negatively predicted it. Sadism was the strongest predictor, adding incremental variance beyond the Big Five and Dark Triad.
Priesemut h & Bigelow (2020)*	Study 1: Canada; Study 2: United States	Mixed industries (retail, education, IT, health care, hospitality, etc.)	Study 1: 111 supervisor–subordinate dyads (supervisors mean age = 33.1, 45.9% female; subordinates	Supervisors as perpetrators of abusive supervision	Field studies using Critical Incident Technique (CIT). Study 1: multisource	Study 1: student-recruited dyads; 42.5% response. Study 2: supervisors recruited via liaisons; 57.7% response	No explicit funding; no conflicts of interest reported	Abusive supervision reduced supervisors' social worth, lowering task performance &

Table S2 Study Characteristics -full data								
**Study (Author, Year)**	**Country / Region**	**Sector / Setting**	**Sample (n, population, occupation)**	**Role focus (Perpetrator)**	**Study Design**	**Sampling method & Response rate**	**Funding / COI**	**Key finding**
			mean age = 20.3, 45.5% female). Study 2: 160 full-time supervisors (mean age = 40.3, 48.8% female, tenure ≈ 9.6 years)		cross-sectional; Study 2: time-lagged (3-week lag)			OCB**.** Psychopathy moderated the effect: low-psychopathy supervisors suffered more losses (and less likely to continue abuse), high-psychopathy buffered (more likely to persist).
Dåderman et al., 2019	Sweden	Public organizations (municipalities, healthcare, schools, and governmental agencies)	N = 247 employees (occupations included administrators, teachers, healthcare staff, social workers; 63% women, mean age 47 years)	Focus on perpetrators (dark triad traits, bullying behaviour)	Cross-sectional survey	Convenience sampling; response rate not explicitly reported	No funding or conflicts of interest declared	Higher scores on Machiavellianism and psychopathy predicted self-reported perpetration of workplace bullying; narcissism was not significant when controlling for the other dark traits.
Carré, Mueller, Schleicher, & Jones (2018)	United States	General working population (via Amazon MTurk)	n = 559 recruited, final N = 481 after attention check; mean age 36.1 years; 61.3%	Employees as potential perpetrators of workplace deviance and	Cross-sectional survey	convenience sampling via Amazon MTurk; response rate not reported (attrition	No specific funding or COI disclosed	Both the Triarchic Psychopathy Model (meanness,

Table S2 Study Characteristics -full data								
**Study (Author, Year)**	**Country / Region**	**Sector / Setting**	**Sample (n, population, occupation)**	**Role focus (Perpetrator)**	**Study Design**	**Sampling method & Response rate**	**Funding / COI**	**Key finding**
			female; occupations varied (e.g., MTurk 13.6%, education 11.1%, sales/marketing 9.0%, healthcare 8.2%, finance 5.7%, etc.); majority White (80%)	sexual harassment		noted through failed attention checks)		disinhibition) and SRP-SF (callous affect) significantly predicted workplace deviance and sexual harassment proclivity. Higher psychopathy traits were associated with higher likelihood of engaging in deviant workplace behaviours
Pilch & Turska, 2015 (*J Bus Ethics*)	Poland	Mixed organisations (private sector 66%; public 34%)	n = 117 employees; 52 men, 65 women; age M = 38 (20–55); 47% university educated; occupations included subordinates (66%) and supervisors (34%)	Both perpetrators and victims examined; focus includes bully-victims (perpetrator + target)	Both perpetrators and victims examined; focus includes bully-victims (perpetrator + target)	Cross-sectional, self-report survey	Not specified (no funding/COI declaration in article)	Machiavellianism significantly predicted bullying others (β = .31, p = .001). Bullies and bully-victims scored higher on Machiavellianism than victims or non-involved. Being bullied was

Table S2 Study Characteristics -full data								
**Study (Author, Year)**	**Country / Region**	**Sector / Setting**	**Sample (n, population, occupation)**	**Role focus (Perpetrator)**	**Study Design**	**Sampling method & Response rate**	**Funding / COI**	**Key finding**
								negatively related to perceptions of clan and adhocracy cultures, and positively related to hierarchy culture. Machiavellianism moderated the relationship between organisational culture and bullying
Wang & Jiang, 2014	Wang & Jiang, 2014	Adult school employees, diverse industries (HR, sales, etc.)	n = 403 full-time employees (95 men, 308 women), M age = 26.0 yrs, M tenure = 5.1 yrs	Subordinates (employees) as potential perpetrators of deviance toward supervisors	Cross-sectional, two-wave survey (1-week apart)	Convenience sampling; response rate not reported	Convenience sampling; response rate not reported	Narcissism was negatively related to perceptions of abusive supervision (r = –.11). Abusive supervision was positively related to deviance toward supervisors (r = .35). Narcissism moderated this link, with

Table S2 Study Characteristics -full data								
**Study (Author, Year)**	**Country / Region**	**Sector / Setting**	**Sample (n, population, occupation)**	**Role focus (Perpetrator)**	**Study Design**	**Sampling method & Response rate**	**Funding / COI**	**Key finding**
								stronger abusive supervision → deviance effects among high-narcissism employees
Burton & Hoobler, 2011*	USA (Midwestern, Southern, Western regions)	Various sectors (management, financial services, science, engineering, healthcare, sales, operations)	Final N = 262 (MBA students working full-time + their coworkers; 44.4% female, mean age 33.4 years, avg. 2.5 years with supervisor)	Subordinates' aggression in response to abusive supervision	Cross-sectional survey, moderated-mediation design	Cross-sectional survey, moderated-mediation design	Not reported	Abusive supervision predicted subordinates' aggression via **interactional justice**; narcissism moderated the justice–aggression link. High-narcissism employees were more aggressive when perceiving injustice, supporting a justice-based moderated-mediation model
Kiazad, Restubog, Zagenczyk,	Australia (Sydney & Brisbane)	Mixed business sectors (finance, manufacturing, healthcare,	92 supervisor–subordinate dyads; subordinates:	Supervisors (Machiavellianism)	Cross-sectional dyadic survey	Research assistants approached ~250 employees; matched surveys from 92	Supported by Australian Research Council Grant	Supervisors' Machiavellianism positively related to subordinates'

Table S2 Study Characteristics -full data								
**Study (Author, Year)**	**Country / Region**	**Sector / Setting**	**Sample (n, population, occupation)**	**Role focus (Perpetrator)**	**Study Design**	**Sampling method & Response rate**	**Funding / COI**	**Key finding**
Kiewitz, & Tang (2010)	Philippines (two large financial institutions)	HR/consulting, public service, legal, retail, education, publishing, mining/engineering)<br><br>Banking sector (fraud & theft seminar course)	51.1% male, mostly aged 20–34, mean tenure 36 months; supervisors: 59.4% male, mostly 35+ yrs, mean tenure 72 months<br><br>200 supervisor–subordinate dyads; subordinates: 67.5% female, mean age 30, mean tenure with supervisor 3.6 years; supervisors: 51% male, 66% aged 31+, 61.5% with ≥6 years tenure	Supervisors (Machiavellianism)	Longitudinal (2-wave, 3-month lag) dyadic survey	dyads (subordinate response 42.8%, supervisor response 36.8%)<br><br>Distributed during training seminar; initial surveys to 300 employees/supervisors, final matched dyads = 200	DP1094023 (to Restubog)<br><br>Supported by Australian Research Council Grant DP1094023 (to Restubog)	perceptions of abusive supervision; effect mediated by authoritarian leadership perceptions.<br><br>Replicated Study 1 in a collectivist context. Supervisor Machiavellianism predicted abusive supervision via authoritarian leadership. Subordinates' low organization-based self-esteem amplified this relationship.
Pullen & Rhodes, 2008*	United Kingdom (UK)	Corporate leadership / organisational case study (gendered	Not quantitative; qualitative interpretive study of organisational leaders (n not specified in	Leaders with narcissistic identity work (focus on narcissistic	Qualitative case-based, interpretive analysis	Qualitative case-based, interpretive analysis	Not explicitly reported; no COI stated	Leadership identity work is framed as narcissistic and gendered; "It's all about me"

Table S2 Study Characteristics -full data								
**Study (Author, Year)**	**Country / Region**	**Sector / Setting**	**Sample (n, population, occupation)**	**Role focus (Perpetrator)**	**Study Design**	**Sampling method & Response rate**	**Funding / COI**	**Key finding**
		leadership contexts	numeric sense, focused on narrative and textual analysis)	leadership as perpetrator role)				captures how narcissism functions in leaders' self-construction, potentially enabling abusive or self-centred organisational practices.

* Not meta-eligible
