# Supplementary material for: Dark Triad traits and workplace bullying: a systematic review and meta-analysis of personality, power, and psychosocial safety
Source: Front Psychol. 2026 Mar 4;17:1738277. doi: 10.3389/fpsyg.2026.1738277 (PMC12995606; doi:10.3389/fpsyg.2026.1738277)
Supplement: Supplementary file 4 [file Data_Sheet_4.pdf]

Table 2Sb. Study Outcomes

Study (Author, Year)	Country / Sector / Setting	Sample (n, population, demographics)	Study aims/hypothesis	Traits / Exposures (instrument, α)	Bullying Perpetration Outcomes (instrument, α, chronicity)	Other Variables (mediators, moderators, confounders)	Role / Target clarity	Design	Analysis	Effect Sizes (β / OR / r + 95% CI)	Effect Direction	Common-method risk	Context moderators	Replicability / Method Notes
Sheng et al.,	China / Hospital (nursing sector)	292 nurses (93% female, mean age 27.3, tenure 3.6 yrs); 50 nurse supervisors (76% female, mean age 38.5, tenure 1.3 yrs)	Examine whether supervisors' narcissism and sleep patterns moderate the relationship between subordinates' counterproductive work behaviours (CWB) and abusive supervision.	Exposure:Subordinate CWB (Bennett & Robinson, 2000; α reported)	Abusive supervision (10-item Aryee et al., 2008, from Tepper, 2000; subordinate-rated; α = .93)	Moderator:Supervisor narcissism (validated scale, α not reported); Moderators:Sleep quality & sleep quantity	Perpetrators = supervisors; Targets = subordinate nurses	Time-lagged survey, 2 waves, 1-month lag	Hierarchical regression with interaction terms	Subordinate CWB → abusive supervision (β significant, p < .01). Supervisor narcissism significantly moderated the relationship (γ = 4.30, p < .01). Simple slopes: high narcissism – CWB significantly predicted abusive supervision (γ = 3.12, p < .001); low narcissism – CWB non-significant (γ = 1.84, p = .06). Sleep quality buffered effect; sleep quantity non-significant.	+ for narcissism moderator; − for sleep quality; null for sleep quantity	Partial bias (self-report for narcissism; subordinates rated abusive supervision); time-lag reduced bias	Chinese healthcare (hospital-based)	Strengths: validated scales, multi-source, moderation tested with slopes; Limitations: single hospital, gender skew, narcissism α not reported

Table 2Sb. Study Outcomes

Study (Author, Year)	Country / Sector / Setting	Sample (n, population, demographics)	Study aims/hypothesis	Traits / Exposures (instrument, α)	Bullying Perpetration Outcomes (instrument, α, chronicity)	Other Variables (mediators, moderators, confounders)	Role / Target clarity	Design	Analysis	Effect Sizes (β / OR / r + 95% CI)	Effect Direction	Common-method risk	Context moderators	Replicability / Method Notes
Jang, Kim & Lee, 2025	Republic of Korea(Tertiary hospital ICU)	47 ICU nurses (21 intervention, 26 control; intervention all female; ≥3 months employment)	To examine the relationship between nursing managers' Dark Triad personality traits (narcissism, Machiavellianism, psychopathy) and their abusive supervision behaviors in Korean	Pathological narcissism (Korean PNI; α reported); interpersonal cognitive distortions; shame; guilt	Workplace bullying perpetration & victimisation (Korean NAQ-R + NAQ-P)	Intervention: Mentalization-Based Art Psychotherapy (MBAP); mediators: shame, guilt, mentalisation	Perpetrators = nurses; Targets = peers/colleagues	Quasi-experimental, 2-group pretest–posttest	ANCOVA comparing intervention vs control	MBAP intervention significantly reduced narcissistic vulnerability (p=0.026), interpersonal cognitive distortions(p=0.022–0.025), and shame (p=0.002); increased mentalization (p=0.004) and guilt (p=0.010). No significant change in bullying perpetration/victimization scores (though downward trends observed).MBAP ↓ narcissistic vulnerability (p = .026), ↓ cognitive	− for vulnerability/cognitive distortions; null trend for bullying	Self-report risk; small N; intervention context reduces bias	Korean ICU context	Strengths: intervention tested, validated scales, ethics approved; Limitations: small N, all-female intervention group, short timeframe

Table 2Sb. Study Outcomes

Study (Author, Year)	Country / Sector / Setting	Sample (n, population, demographics)	Study aims/hypothesis	Traits / Exposures (instrument, α)	Bullying Perpetration Outcomes (instrument, α, chronicity)	Other Variables (mediators, moderators, confounders)	Role / Target clarity	Design	Analysis	Effect Sizes (β / OR / r + 95% CI)	Effect Direction	Common-method risk	Context moderators	Replicability / Method Notes
			hospital settings.<br><br>To identify which of the Dark Triad traits most strongly predict abusive supervision as perceived by subordinate nurses.<br><br>Each Dark Triad trait → abusive							distortions (p = .022–.025), ↓ shame (p = .002); ↑ mentalisation (p = .004), ↑ guilt (p = .010). No significant change in bullying perpetration/victimisation scores (downward trend only)				

Table 2Sb. Study Outcomes

Study (Author, Year)	Country / Sector / Setting	Sample (n, population, demographics)	Study aims/hypothesis	Traits / Exposures (instrument, α)	Bullying Perpetration Outcomes (instrument, α, chronicity)	Other Variables (mediators, moderators, confounders)	Role / Target clarity	Design	Analysis	Effect Sizes (β / OR / r + 95% CI)	Effect Direction	Common-method risk	Context moderators	Replicability / Method Notes
			supervision: Nursing managers higher in narcissism, Machiavellianism, and psychopathy will display higher levels of abusive supervision.<br><br>Differential strength: Among the three traits, narcissism will have											

Table 2Sb. Study Outcomes

Study (Author, Year)	Country / Sector / Setting	Sample (n, population, demographics)	Study aims/hypothesis	Traits / Exposures (instrument, α)	Bullying Perpetration Outcomes (instrument, α, chronicity)	Other Variables (mediators, moderators, confounders)	Role / Target clarity	Design	Analysis	Effect Sizes (β / OR / r + 95% CI)	Effect Direction	Common-method risk	Context moderators	Replicability / Method Notes
			the strongest positive association with abusive supervision (based on prior theory and East Asian hierarchical culture).<br><br>Control variables: The effects will hold even when controlling for											

Table 2Sb. Study Outcomes

Study (Author, Year)	Country / Sector / Setting	Sample (n, population, demographics)	Study aims/hypothesis	Traits / Exposures (instrument, α)	Bullying Perpetration Outcomes (instrument, α, chronicity)	Other Variables (mediators, moderators, confounders)	Role / Target clarity	Design	Analysis	Effect Sizes (β / OR / r + 95% CI)	Effect Direction	Common-method risk	Context moderators	Replicability / Method Notes
			demographic/workplace characteristics (age, tenure, etc.).											
Braun et al., 2024	Germany (Study 1–2); UK (Study 3) / organisatio	Study 1: N=320 supervisors. Study 2: N=326 supervisors. Study 3: N=292 supervisors (age 19–66, M=34.6;	Test whether leaders high in vulnerable narcissism engage in abusive supervision via shame, and whether attributional conditions	Vulnerable narcissism (Pathological Narcissism Inventory – Short Form; α ≈ .89). Grandiose	Abusive supervision (Tepper scale adapted to intentions; α ≈ .91–. 92). Outcome	Mediator: shame. Moderator: attributional condition (internal vs external vs control). Control variable:	Perpetrator: supervisors/leaders. Target: subordinates (hypothetical or recalled).	Study 1: correlational survey; Study 2: experimental scenario; Study 3: experi	□ S1: “Hierarchical regression; PROCESS Model 4 (mediation).”	Study Direct: Vulnerable narcissism → abusive supervision B = 0.258, 95% CI [0.196, 0.320], *p* < .001.Path a: Vulnerable narcissism → internal attribution B = 0.161, 95% CI [0.035, 0.287], *p* < .05. Path b: Internal attribution → abusive supervision B = 0.064,	Positive (vulnerable narcissism ↑ abusive supervision; mediated	Self-report (intentions) → risk of bias; mitigated by experimental manipulations.	German / UK leadership context. Attribution conditions as conte	High internal validity from experiments. External validity limited (self-selected supervisors, Western context). Reliability of attribution scale in Study 1 lower than desired.

Table 2Sb. Study Outcomes

Study (Author, Year)	Country / Sector / Setting	Sample (n, population, demographics)	Study aims/hypothesis	Traits / Exposures (instrument, α)	Bullying Perpetration Outcomes (instrument, α, chronicity)	Other Variables (mediators, moderators, confounders)	Role / Target clarity	Design	Analysis	Effect Sizes (β / OR / r + 95% CI)	Effect Direction	Common-method risk	Contextual moderators	Replicability / Method Notes
	nal & leadership settings	~5.5 yrs supervisory experience; roles: team leaders, dept managers, area managers, TMT).	alter this indirect process. Hypothesis 1. Leaders' vulnerable narcissism relates posi-tively to abusive supervision. Hypothesis 2. Internal attribution of failure mediates the positive relationship between leaders'	narcissism also measured.	measured as self-reported abusive supervision behaviours in scenarios / recall tasks.	grandiose narcissism.		mental recall (manipulation-of-mediator).	S2: "PROCESS Model 8 (moderated mediation; internal/external/control; 5,000 bootstraps)."<br><br>S3: "PROCESS Model	95% CI [0.011, 0.118], $p < .05$. (Indirect effect reported via Model 4; numeric total not printed in text extract—paths a & b above are provided.)<br><br>Study 2: Direct (VN → abusive supervision intentions) by condition: Internal attribution: B = 0.290, SE = 0.075, 95% CI [0.142, 0.438]. External attribution: B = 0.287, SE = 0.076, 95% CI [0.139, 0.436]. Control: B = 0.424, SE = 0.075, 95%	by shame).		xtual moderators.	

Table 2Sb. Study Outcomes

Study (Author, Year)	Country / Sector / Setting	Sample (n, population, demographics)	Study aims/hypothesis	Traits / Exposures (instrument, α)	Bullying Perpetration Outcomes (instrument, α, chronicity)	Other Variables (mediators, moderators, confounders)	Role / Target clarity	Design	Analysis	Effect Sizes (β / OR / r + 95% CI)	Effect Direction	Common-method risk	Context moderators	Replicability / Method Notes
			vulnerable narcissism and abusive supervision. Hypothesis 3. The relationship between leaders’ vulnerable narcissism and abusive supervision is mediated by internal attribution of failure and shame in a serial fashion.						8 (moderated mediation; two dummy variables; 5,000 bootstraps).”	CI [0.277, 0.571].Conditional indirect (VN → shame → abusive supervision intentions): Internal attribution: B = 0.029, 95% CI [0.001, 0.068] (sig). External attribution: B = 0.064, 95% CI [0.012, 0.108] (sig). Control: B = 0.058, 95% CI [0.015, 0.103] (sig).<br><br>Study 3: Direct (across conditions combined): Vulnerable narcissism → abusive supervision B = 0.149, SE = 0.059, 95%				

Table 2Sb. Study Outcomes

Study (Author, Year)	Country / Sector / Setting	Sample (n, population, demographics)	Study aims/hypothesis	Traits / Exposures (instrument, α)	Bullying Perpetration Outcomes (instrument, α, chronicity)	Other Variables (mediators, moderators, confounders)	Role / Target clarity	Design	Analysis	Effect Sizes (β / OR / r + 95% CI)	Effect Direction	Common-method risk	Context moderators	Replicability / Method Notes
										CI [0.033, 0.265], $p < .05$.Conditional indirect (VN → shame → abusive supervision):Internal attribution: B = 0.053, 95% CI [0.005, 0.122] (sig).External attribution: B = 0.038, 95% CI [0.009, 0.077] (sig).Control: B = 0.007, 95% CI [−0.029, 0.049] (ns).				
Jang et al., 2023	Republic of Korea / ICU	N = 416 ICU nurses; 91.3% female; mean age 30.8	To examine how personality traits (including dark personality facets)	Dark personality / Dark Triad (Short Dark	Workplace bullying—Perpetrator: Negative	Covariates entered from characteristics & correlati	Self-report of victim and perpetrator experiences among	Cross-sectional online survey (July 2022)	Descriptives; t-tests/ANOVA; Pearso	Perpetrator model (final, adjusted): Dark personality β = .48, p < .001; Mentalization β = −.17, p < .001; Education level β = .15, p	Dark personality → positive; ment	Single-source, cross-sectional self-report (risk of CMV and temporal ambiguity).	CU setting in South Korea; org	Large N; convenience sample; validated bullying instruments; clear regression

Table 2Sb. Study Outcomes

Study (Author, Year)	Country / Sector / Setting	Sample (n, population, demographics)	Study aims/hypothesis	Traits / Exposures (instrument, α)	Bullying Perpetration Outcomes (instrument, α, chronicity)	Other Variables (mediators, moderators, confounders)	Role / Target clarity	Design	Analysis	Effect Sizes (β / OR / r + 95% CI)	Effect Direction	Common-method risk	Context moderators	Replicability / Method Notes
	nursing in tertiary hospitals	2 (SD 5.47); convenience online recruitment via hospital groupware & nurse communities	and mentalization relate to workplace bullyingamong ICU nurses, after controlling for organizational culture	Triad; 3 factors: narcissism, Machiavellianism, psychopathy), 5-point scale; α ≈ .80–.87 (reported range across subscales). Perfectionistic Self-Presentation (17-	Acts Questionnaire–Perpetrator (NAQ-P), adapted from NAQ-R; Cronbach's α = .97 (original and this study). Work	ons; organizational culture (Positive Nursing Organizational Culture Measurement Tool; 26 items; α = .96 in this study). Other covariates included educati	ICU nurses (no supervisor–subordinate dyads)		n correlations; multiple regression (enter method). Diagnostics reported: VIF 1.04–3.62, Durbin–Watson = 1.90,	= .023; Perfectionistic self-presentation β = −.13, p = .007; Subjective health status β = .13, p = .001. 95% CIs NR in the text; model Adjusted R^2 = .41	alization → negative; education → positive; perfectionistic self-presentation → negative; subje	Assumption checks reported; no time lag or multi-source	anizational culture statistically controlled in models	diagnostics. No CIs reported for β; results suitable for meta-analysis using β + p (or request SEs from authors if needed)

Table 2Sb. Study Outcomes

Study (Author, Year)	Country / Sector / Setting	Sample (n, population, demographics)	Study aims/hypothesis	Traits / Exposures (instrument, α)	Bullying Perpetration Outcomes (instrument, α, chronicity)	Other Variables (mediators, moderators, confounders)	Role / Target clarity	Design	Analysis	Effect Sizes (β / OR / r + 95% CI)	Effect Direction	Common-method risk	Context moderators	Replicability / Method Notes
				item Korean version; subscales: self-promotion, nondisplay, nondisclosure), α NR here. Mentalization (scale name not cleanly visible in text; α reported	place bullying—Victim: NAQ-R (22 items), α = .93 (orig)/ .96(Korean version)	on, subjective health, etc.			K–S Z = 0.05, p = .269; Adjusted R^2 = .41 for perpetrator model		ctive health → positive (toward perpetration score)			

Table 2Sb. Study Outcomes

Study (Author, Year)	Country / Sector / Setting	Sample (n, population, demographics)	Study aims/hypothesis	Traits / Exposures (instrument, α)	Bullying Perpetration Outcomes (instrument, α, chronicity)	Other Variables (mediators, moderators, confounders)	Role / Target clarity	Design	Analysis	Effect Sizes (β / OR / r + 95% CI)	Effect Direction	Common-method risk	Context moderators	Replicability / Method Notes
				in paper's measures section but not retrievable here → NR)										
Feng et al., 2023	Study 1: USA, cross-industry MTurk. Stu	Study 1: n=355 managers (avg. age 38; 56% male; supervising ≥2 subordinates). Study	Test whether leader Machiavellianism predicts abusive supervision via leader–direct supervisor guanxi (LDG). Hypotheses	Leader Machiavellianism (Mach IV, Christie & Geis, 1970; 20 items, α=.87 Study 1; α=.85	Abusive supervision: Study 1 leader self-report (Tepper, 2000; 15 items,	Mediator: Leader–direct supervisor guanxi (Chen et al., 2009, α=.92). Moderator: Team -	Perpetrator role: leaders/supervisors. Targets: subordinates (Study 2).	Study 1: Two-wave, time-lagged, single-source survey. Study 2: Two-wave, multisource,	Study 1: OLS regression + bootstrapped indirect effects. Study 2: Multilevel structu	Study 1: Machiavellianism → LDG: β=.36, p<.001; LDG → abusive supervision: β=.21, p<.01; Indirect effect: β=.08, 95% CI [.03, .15]. Study 2: Machiavellianism → LDG: γ=.28, p<.001; LDG → abusive supervision: γ=.19, p<.01; Indirect	Positive associations: Machiavellianism ↑ → abusive supervisio	Study 1: Single-source (leader-rated) → potential CMV. Study 2: Multi-source (leader & subordinate) reduces CMV.	Cultural context moderators: Guanxi (LDG/TMG) uniqu	High replicability: Two independent samples (US & China), multisource, consistent results. Notes: high reliabilities (α>.85); strong sample size.

Table 2Sb. Study Outcomes

Study (Author, Year)	Country / Sector / Setting	Sample (n, population, demographics)	Study aims/hypothesis	Traits / Exposures (instrument, α)	Bullying Perpetration Outcomes (instrument, α, chronicity)	Other Variables (mediators, moderators, confounders)	Role / Target clarity	Design	Analysis	Effect Sizes (β / OR / r + 95% CI)	Effect Direction	Common-method risk	Context moderators	Replicability / Method Notes
	dy 2: China, state-owned power company, 13 branches.	2: *T1:* 1,347 subordinates & 298 leaders; *T2:* 1,252 subordinates & 273 leaders; intact teams; demographics reported (gender, age, tenure).	: (H1) Machiavellianism → LDG → abusive supervision. (H2) Team-member guanxi (TMG) moderates LDG→abuse. (H3) Abusive supervision reduces team outcomes (voice, OCBI).	Study 2).	α=.95). Study 2 subordinate report aggregated to team-level (Tepper, 2000; α=.95). Chronicity: two-wave lagged (4 weeks).	member guanxi (TMG, α=.91). Outcomes: Team voice (Liang et al., 2012, α=.91) & OCBI (Lee & Allen, 2002, α=.92). Controls: leader age, gender, tenure,		multilevel field study.	ral equation modelling (MSEM).	effect: γ=.05, 95% CI [.02, .10]. Moderator (TMG): γ=.11, p<.01 (strengthens LDG→abuse). Abuse → team voice: γ=−.22, p<.001; abuse → OCBI: γ=−.18, p<.001.	n ↑. Negative associations: Abuse ↑ → team outcomes ↓.		ely Chinese, but theorised to extend to relationship-oriented contexts.	

Table 2Sb. Study Outcomes

Study (Author, Year)	Country / Sector / Setting	Sample (n, population, demographics)	Study aims/hypothesis	Traits / Exposures (instrument, α)	Bullying Perpetration Outcomes (instrument, α, chronicity)	Other Variables (mediators, moderators, confounders)	Role / Target clarity	Design	Analysis	Effect Sizes (β / OR / r + 95% CI)	Effect Direction	Common-method risk	Context moderators	Replicability / Method Notes
						education, negative affectivity.								
Preston et al	USA / community employees recruited via MTurk	n = 331 employed adults (51.1% female, mean age 37, range 19–79, diverse occupations; majority White, 62%)	To test how psychopathic traits (boldness, meanness, disinhibition, antisocial behavior) relate to counterproductive workplace behaviours (CWB), controlling for other	Triarchic Psychopathy Measure (TriPM): Boldness (α = .87), Meanness (α = .93), Disinhibition (α = .90). Elemental	Counterproductive Work Behavior Checklist (CWB-C; Spector et al., 2006); 32 items; α = .96	Covariates: age, gender, education, race; employment sector; Big Five traits controlled in supplementary models.	Role: employee self-report as potential perpetrator (CWB behaviors). Targets: coworkers / organization.	Cross-sectional, online survey (single wave).	Hierarchical regression; zero-order correlations; interaction tests; supplementary robustness	Key betas (Table 3): Disinhibition → total CWB $\beta = .44$, $p < .001$. Antisocial → CWB $\beta = .28$, $p < .001$. Meanness → interpersonal CWB $\beta = .19$, $p < .01$. Boldness → organizational CWB $\beta = -.12$, $p < .05$ (protective). R^2 for total CWB = .40. 95% CIs not reported (p-values given).	Key betas (Table 3): Disinhibition → total CWB $\beta = .44$, $p < .001$. Antis	High CMV risk: all measures via self-report, same time.	No explicit contextual moderators; effects robust across demo	Solid reliability of psychopathy & CWB measures; large, heterogeneous adult sample; but MTurk limits generalisability. Single-source design = replication should use multi-source/longitudinal data.

Table 2Sb. Study Outcomes

Study (Author, Year)	Country / Sector / Setting	Sample (n, population, demographics)	Study aims/hypothesis	Traits / Exposures (instrument, α)	Bullying Perpetration Outcomes (instrument, α, chronicity)	Other Variables (mediators, moderators, confounders)	Role / Target clarity	Design	Analysis	Effect Sizes (β / OR / r + 95% CI)	Effect Direction	Common-method risk	Context moderators	Replicability / Method Notes
			dark traits. Hyp: Disinhibition & Antisocial → ↑ CWB; Boldness → adaptive; Meanness → interpersonal CWB.	Psychopathy Assessment (EPA): Antisocial behavior subscale (α = .82).	overall; subscales: organizational CWB (α = .94), interpersonal CWB (α = .92). Time frame: past 12 months.				checks.		ocial → CWB $\beta = .28$, $p < .001$. Meanness → interpersonal CWB $\beta = .19$, $p < .01$. Bold		graphics.	

Table 2Sb. Study Outcomes

Study (Author, Year)	Country / Sector / Setting	Sample (n, population, demographics)	Study aims/hypothesis	Traits / Exposures (instrument, α)	Bullying Perpetration Outcomes (instrument, α, chronicity)	Other Variables (mediators, moderators, confounders)	Role / Target clarity	Design	Analysis	Effect Sizes (β / OR / r + 95% CI)	Effect Direction	Common-method risk	Context moderators	Replicability / Method Notes
											ness → organizational CWB β = −.12, $p < .05$ (protective). R^2 for total CWB = .40 . 95% CIs not			

Table 2Sb. Study Outcomes

Study (Author, Year)	Country / Sector / Setting	Sample (n, population, demographics)	Study aims/hypothesis	Traits / Exposures (instrument, α)	Bullying Perpetration Outcomes (instrument, α, chronicity)	Other Variables (mediators, moderators, confounders)	Role / Target clarity	Design	Analysis	Effect Sizes (β / OR / r + 95% CI)	Effect Direction	Common-method risk	Context moderators	Replicability / Method Notes
											reported (p-values given).			
Fernández-del-Río, Ramos-Villagrasa, & Escartín (2021)	Spain / mixed organizations (employees across	n = 613 employees; 54% female; mean age 38.8 yrs (SD = 14.1); mean tenure 8.4 yrs (SD = 10.1)	Test whether Dark Tetrad traits (Machiavellianism, narcissism, psychopathy, sadism) predict workplace bullying perpetration,	Dark Triad Dirty Dozen (DTDD; Jonason & Webster, 2010): Machiavellianism, narcissism,	Negative Acts Questionnaire–Revised (NAQ-R; Einarsen et al., 2009)	Controlled for Big Five traits; demographic covariates (age, gender, tenure) included. No mediators/moder	Focus on perpetration (self-reported bullying acts)	Cross-sectional, self-report paper survey	Hierarchical regression (stepwise); incremental variance tested with ΔR^2	β coefficients (Table 2 & 3 in paper): Sadism $\beta = .28$, $p < .001$; Narcissism $\beta = .11$, $p < .05$; Machiavellianism $\beta = .09$, ns; Psychopathy $\beta = .07$, ns. Incremental R^2 (Dark Tetrad over Big Five) = .07.	Positive: Sadism & narcissism significantly predicted perpetration;	High CMV risk: self-report, same source/time; Harman's test not reported.	Spanish workplace culture context (collectivist values, labou	Good psychometric reliability; strong sample size; incremental validity analysis improves robustness. Cross-sectional = limits causal inference.

Table 2Sb. Study Outcomes

Study (Author, Year)	Country / Sector / Setting	Sample (n, population, demographics)	Study aims/hypothesis	Traits / Exposures (instrument, α)	Bullying Perpetration Outcomes (instrument, α, chronicity)	Other Variables (mediators, moderators, confounders)	Role / Target clarity	Design	Analysis	Effect Sizes (β / OR / r + 95% CI)	Effect Direction	Common-method risk	Context moderators	Replicability / Method Notes
	multiple sectors)		controlling for Big Five; hypothesised that sadism adds incremental variance.	psychopathy (α ≈ .70–.84 in study). Short Sadistic Impulse Scale (SSIS; O'Meara et al., 2011), α = .74. Big Five: Ten-Item Personality Inventory (TIPI;	perpetration version; 22 items; α = .90. Frequency past 6 months.	ators tested.					Machiavellianism and psychopathy nonsignificant when controlling for Big Five.		r protections) may shape bullying norms.	

Table 2Sb. Study Outcomes

Study (Author, Year)	Country / Sector / Setting	Sample (n, population, demographics)	Study aims/hypothesis	Traits / Exposures (instrument, α)	Bullying Perpetration Outcomes (instrument, α, chronicity)	Other Variables (mediators, moderators, confounders)	Role / Target clarity	Design	Analysis	Effect Sizes (β / OR / r + 95% CI)	Effect Direction	Common-method risk	Context moderators	Replicability / Method Notes
				Gosling et al., 2003), α = .68–.74.										
Priesemuth & Bigelow (2020)*	Study 1: Canada; Study 2: U.S. (mixed industries:	Study 1: 111 supervisor–subordinate dyads (supervisors: M age 33.1, 45.9% female; subordinates: M age	To test whether abusive supervision has negative consequences for supervisors themselves (via social worth depletion), and whether psychopathy	Supervisor psychopathy (Triarchic Psychopathy Measure – TriPM, α ≈ .84); Abusive supervision incident	Abusive supervision (Critical Incident Technique – self-reported abusive acts within	Mediator: Perceived social worth; Outcomes: task performance & OCB. Moderator: Psychopathy	Mediator: Perceived social worth; Outcomes: task performance & OCB. Moderator: Psychopathy	Study 1: Cross-sectional, multisource CIT; Study 2: Time-lagged (3 weeks)	Study 1: Regression & mediation (PROCESS). Study 2: Moderated mediation	Study 1: Abusive supervision → ↓ social worth (B = –0.36, $p < .01$); Social worth → ↓ performance (B = 0.22, $p < .05$). Study 2: Interaction abusive supervision × psychopathy → social worth (B = 0.15, $p < .05$). Conditional effects: low psychopathy = strong negative effect; high psychopathy = non-significant.	Negative direction: abusive supervision harms social worth & outco	Study 1 partially reduces CMV via multisource (sup. vs sub.); Study 2 single-source risk but time-lag helps	Context moderators: Psychopathy moderates pathway	Well-designed with CIT across two samples; replication strength from time-lagged design; both studies converge on finding that psychopathy shields abusive supervisors from negative consequences

Table 2Sb. Study Outcomes

Study (Author, Year)	Country / Sector / Setting	Sample (n, population, demographics)	Study aims/hypothesis	Traits / Exposures (instrument, α)	Bullying Perpetration Outcomes (instrument, α, chronicity)	Other Variables (mediators, moderators, confounders)	Role / Target clarity	Design	Analysis	Effect Sizes (β / OR / r + 95% CI)	Effect Direction	Common-method risk	Context moderators	Replicability / Method Notes
	retail, education, IT, healthcare, etc.)	20.3, 45.5% female). Study 2: 160 full-time supervisors (M age 40.3, 48.8% female, tenure ≈ 9.6 yrs)	moderates these effects	s (self-report, CIT coding)	past 6 months; no α as CIT coded events)						mes, but high psychopathy buffers			
Dåderman et al., 2019	Sweden / Public organi	N = 247 employees; 63% women; mean age 47 (range	To examine whether Machiavellianism, narcissism, and psychopath	Dark Triad Dirty Dozen (Jonason & Webster,	Negative Acts Questionnaire–Revise	Gender, age, education included as	Perpetrator focus (self-reported bullying behaviour)	Cross-sectional survey	Multiple linear regression; hierarchical	Machiavellianism → bullying β = .29, p < .001; Psychopathy → bullying β = .21, p < .01; Narcissism → bullying β = .07 (ns). R^2 = .17.	Positive effects for Machiavellianis	Self-report design (traits + bullying); possible CMV inflation	Swedish municipal/healthcare/scho	Replicable; standard instruments; convenience sample; cross-sectional limits causal inference

Table 2Sb. Study Outcomes

Study (Author, Year)	Country / Sector / Setting	Sample (n, population, demographics)	Study aims/hypothesis	Traits / Exposures (instrument, α)	Bullying Perpetration Outcomes (instrument, α, chronicity)	Other Variables (mediators, moderators, confounders)	Role / Target clarity	Design	Analysis	Effect Sizes (β / OR / r + 95% CI)	Effect Direction	Common-method risk	Context moderators	Replicability / Method Notes
	zations (municipalities, healthcare, schools, government agencies)	21–65); occupations: administrators, teachers, healthcare staff, social workers	y predict self-reported workplace bullying perpetration	2010); 12 items, α ≈ .73–.84 (reported in original, this study confirms acceptable reliabilities for each subscale)	d (NAQ-R), perpetrator version; 22 items, α = .91; frequency past 6 months (≥2 negative acts weekly = bullying)	covariates			regression models		m and psychopathy; no effect for narcissism		ol context	

Table 2Sb. Study Outcomes

Study (Author, Year)	Country / Sector / Setting	Sample (n, population, demographics)	Study aims/hypothesis	Traits / Exposures (instrument, α)	Bullying Perpetration Outcomes (instrument, α, chronicity)	Other Variables (mediators, moderators, confounders)	Role / Target clarity	Design	Analysis	Effect Sizes (β / OR / r + 95% CI)	Effect Direction	Common-method risk	Context moderators	Replicability / Method Notes
Carré et al., 2018	United States / General workforce (via MTurk)	n = 559 recruited, final N = 481 (61.3% female, mean age 36.1, occupations across multiple sectors, 80% White)	To compare the predictive validity of the Triarchic Psychopathy Measure (TriPM) and the Self-Report Psychopathy–Short Form (SRP-SF) for workplace deviance and sexual harassment proclivity	**Triarchic Psychopathy Measure (TriPM)** – Meanness (α = .89), Disinhibition (α = .87), Boldness (α = .84); **SRP-SF** – Callous affect, erratic	**Workplace Deviance Scale (Bennett & Robinson, 2000)**: Organizational deviance (α = .93), Interpersonal deviance (α = .94);	Demographics (gender, age, race) controlled; also compared incremental predictive validity across psychopathy models	Perpetrator focus – employees reporting their own deviant/harassing behaviours	Cross-sectional online survey	Hierarchical regression; incremental validity analyses; bivariate correlations	Hierarchical regression; incremental validity analyses; bivariate correlations	Positive direction: higher psychopathy → higher deviance & harassment	Self-report, same-source, cross-sectional; CMV risk high	Online MTurk sample (mostly U.S. adults in diverse occupations)	Replicable with MTurk samples; validated instruments; large sample; limitation = no longitudinal or behavioural validation

Table 2Sb. Study Outcomes

Study (Author, Year)	Country / Sector / Setting	Sample (n, population, demographics)	Study aims/hypothesis	Traits / Exposures (instrument, α)	Bullying Perpetration Outcomes (instrument, α, chronicity)	Other Variables (mediators, moderators, confounders)	Role / Target clarity	Design	Analysis	Effect Sizes (β / OR / r + 95% CI)	Effect Direction	Common-method risk	Context moderators	Replicability / Method Notes
				lifestyle, interpersonal manipulation, criminal tendencies (α = .70–.84)	**Sexual Harassment Proclivity Scale** (α = .88). Outcomes are **self-reported proclivities/perpetration likelihood**(cross-									

Table 2Sb. Study Outcomes

**Study (Author, Year)**	**Country / Sector / Setting**	**Sample (n, population, demographics)**	**Study aims/hypothesis**	**Traits / Exposures (instrument, α)**	**Bullying Perpetration Outcomes (instrument, α, chronicity)**	**Other Variables (mediators, moderators, confounders)**	**Role / Target clarity**	**Design**	**Analysis**	**Effect Sizes (β / OR / r + 95% CI)**	**Effect Direction**	**Common-method risk**	**Context moderators**	**Replicability / Method Notes**
					sectional, no chronicity)									
Pilch & Turska, 2015	Poland / Mixed organisations (private + public sector)	n = 117 employees; 52 men, 65 women; mean age 38 (range 20–55); 47% with university education	Examine the role of Machiavellianism in bullying perpetration and its interplay with organisational culture	**Machiavellianism** – Mach-IV scale (Christie & Geis, 1970), Cronbach's α = .74	**Bullying perpetration** Unethical Behavior Questionnaire (UBQ) (Chudzicka and Makselon-	Organisational culture (Clan, Adhocracy, Market, Hierarchy) via Organizational Culture Assessment Instrument (OCAI);	Clear — perpetrators, victims, bully-victims, and non-involved identified separately	Cross-sectional survey	Hierarchical regression and moderation analysis	Machiavellianism → bullying others: β = .31, p = .001. Bully-victims scored highest on Machiavellianism (M = 108.0) vs non-involved (M = 92.5). Organisational culture significantly related to being bullied: clan/adhocracy (neg.), hierarchy (pos.)	Positive for Machiavellianism predicting bullying perpetration	High (self-report survey; cross-sectional)	Organisational culture moderated Machiavellianism–bullying relationship	Replicable with larger, more representative samples; snowball sampling is a limitation

Table 2Sb. Study Outcomes

Study (Author, Year)	Country / Sector / Setting	Sample (n, population, demographics)	Study aims/hypothesis	Traits / Exposures (instrument, α)	Bullying Perpetration Outcomes (instrument, α, chronicity)	Other Variables (mediators, moderators, confounders)	Role / Target clarity	Design	Analysis	Effect Sizes (β / OR / r + 95% CI)	Effect Direction	Common-method risk	Context moderators	Replicability / Method Notes
					Kowalska 2004).	Machiavellianism tested as moderator								
Wang & Jiang, 2014*	China / Employees across multiple sectors (adult	n = 403 full-time employees (95 men, 308 women), mean age 26 yrs, mean tenure [illegible] yrs	To examine whether narcissism moderates and mediates the relationship between abusive supervision and deviant behavior toward supervisors	**Narcissism** – NPI-16 (Ames et al., 2006), α = .71	**Deviant behavior toward supervisors** – Bennett & Robinson Workplace Devian	Mediator: Perception of abusive supervision (Tepper's 15-item scale, α = .92). Moderator:	Focus is on **subordinates as perpetrators** of deviant behavior toward supervisors	Cross-sectional, two-wave survey (1-week interval to reduce CMV)	Cross-sectional, two-wave survey (1-week interval to reduce CMV)	Abusive supervision → deviance (β = .35, p < .001). Narcissism × abusive supervision interaction → deviance (β = .18, p < .01). Narcissism → lower perception of abusive supervision (β = –.11, p < .05). Indirect + interaction supported "dual role."	Positive: abusive supervision increases deviance; narcissism strengthen	Self-report, though two-wave design reduces CMV	Chinese organisational/cultural context (power distance, supervisor	Replicable; validated measures; limitation: cross-sectional (short lag), convenience sample, cultural specificity

Table 2Sb. Study Outcomes

Study (Author, Year)	Country / Sector / Setting	Sample (n, population, demographics)	Study aims/hypothesis	Traits / Exposures (instrument, α)	Bullying Perpetration Outcomes (instrument, α, chronicity)	Other Variables (mediators, moderators, confounders)	Role / Target clarity	Design	Analysis	Effect Sizes (β / OR / r + 95% CI)	Effect Direction	Common-method risk	Context moderators	Replicability / Method Notes
	school in Beijing)				ce Scale (supervisor-targeted items), α = .89; timeframe = past 6 months	Narcissism.					s this effect (moderator) and decreases perception of abuse (mediator)		– subordinate norms)	
Burton & Hoobler, 2011*	USA / multiple sectors	N = 262 (MBA students working full-time + ≥4 coworke	To test whether **interactional justice mediates** the abusive supervision	**Narcissism** – NPI-16 (Ames et al., 2006), α = .76. **A**	**Subordinate aggression toward superv**	Mediator: **Interactional justice** (Colquitt's scale, α = .93).	Clear: subordinate aggression n = perpetrator	Cross-sectional, multi-source survey (subor	Hierarchical regression; moderated mediat	Abusive supervision → aggression (β = .28, p < .001). Interactional justice mediates this link (indirect effect significant, Sobel test z = 2.14, p < .05).	Positive: more abusive supervisio	Some CMV risk reduced via coworker reports + separate sources	Context: U.S. MBA working adult	Replicable but limited by convenience MBA sampling, snowball coworker design, cross-sectional

Table 2Sb. Study Outcomes

Study (Author, Year)	Country / Sector / Setting	Sample (n, population, demographics)	Study aims/hypothesis	Traits / Exposures (instrument, α)	Bullying Perpetration Outcomes (instrument, α, chronicity)	Other Variables (mediators, moderators, confounders)	Role / Target clarity	Design	Analysis	Effect Sizes (β / OR / r + 95% CI)	Effect Direction	Common-method risk	Context moderators	Replicability / Method Notes
	(management, finance, science, engineering, healthcare, sales, ops)	rs); mean age 33.4 yrs; 44.4% female; avg. 2.5 yrs with supervisor	→ subordinate aggression relationship, and whether **narcissism moderates** the justice–aggression link	**busive supervision** – Tepper's 15-item scale, α = .91	**isor** (adapted Bennett & Robinson interpersonal deviance scale), α = .83; chronicity: workplace deviant behaviours within	Moderator: Narcissism. Covariates: sex, age, tenure, negative affectivity	outcome; abusive supervision = predictor	dinate reports + coworker ratings)	ion (Baron & Kenny approach)	Narcissism moderates justice → aggression (interaction β = .16, p < .05). High narcissism strengthens effect of injustice on aggression	n → more aggression; narcissism amplifies the injustice → aggression pathway		s, diverse industries	

Table 2Sb. Study Outcomes

Study (Author, Year)	Country / Sector / Setting	Sample (n, population, demographics)	Study aims/hypothesis	Traits / Exposures (instrument, α)	Bullying Perpetration Outcomes (instrument, α, chronicity)	Other Variables (mediators, moderators, confounders)	Role / Target clarity	Design	Analysis	Effect Sizes (β / OR / r + 95% CI)	Effect Direction	Common-method risk	Context moderators	Replicability / Method Notes
					past year									
Kiazad et al., 2010	Australia / Mixed industries (finance, manufacturing, healthcare,	92 supervisor–subordinate dyads; subordinates: 51.1% male, mostly 20–34 yrs; mean tenure 36 months; supervisors:	Test whether supervisors' Machiavellianism predicts abusive supervision, mediated by authoritarian leadership perceptions<br><br>Replicate Study 1 in collectivist culture; test moderation	**Supervisor Machiavellianism** – Mach IV scale (Christie & Geis, 1970); $\alpha = .76$<br><br>**Supervisor Machiavellianism** – Mach IV scale	**Abusive supervision (perpetration via subordinate reports)** – Tepper's 15-item Abusive Supervision	Mediator: Authoritarian leadership perceptions; Control variables: subordinate age, gender, tenure, education, organiza	Perpetrator = Supervisor	Cross-sectional dyadic survey<br><br>Longitudinal (2 waves, 3-month lag)	Hierarchical regression & mediation (Baron & Kenny, Sobel test)<br><br>Hierarchical moderated mediation	Machiavellianism → Abusive supervision $\beta = .32$, $p < .01$; Indirect effect via authoritarian leadership $\beta = .24$ (Sobel $z = 2.54$, $p < .05$)<br><br>Machiavellianism → Abusive supervision $\beta = .26$, $p < .01$; Indirect effect via authoritarian leadership $\beta = .19$ ($p < .05$). Moderation: Effect stronger for low OBSE (interaction $\beta = −.21$, $p < .01$)	Positive<br><br>Positive (stronger for low OBSE)	Risk reduced (dyadic, independent supervisor/subordinate reports, mediation tested)<br><br>Reduced risk (time-lag, independent supervisor & subordinate reports)	Western/individualist culture<br><br>Collectivist context (Philippines banking)	Well-powered but cross-sectional; replication in collectivist context (Study 2)<br><br>Replicated cross-cultural validity; stronger evidence than Study 1

Table 2Sb. Study Outcomes

Study (Author, Year)	Country / Sector / Setting	Sample (n, population, demographics)	Study aims/hypothesis	Traits / Exposures (instrument, α)	Bullying Perpetration Outcomes (instrument, α, chronicity)	Other Variables (mediators, moderators, confounders)	Role / Target clarity	Design	Analysis	Effect Sizes (β / OR / r + 95% CI)	Effect Direction	Common-method risk	Context moderators	Replicability / Method Notes
	HR/consulting, public service, legal, retail, education, publishing, min	59.4% male, mostly 35+, mean tenure 72 months<br><br>200 supervisor–subordinate dyads; subordinates: 67.5% female, mean age 30,	by organization-based self-esteem (OBSE)	(Christie & Geis, 1970); α = .76	Scale; α = .95<br><br>**Abusive supervision (perpetration via subordinate reports)** – Tepper's scale; α = .94	tional tenure<br><br>Mediator: Authoritarian leadership; Moderator: Subordinate OBSE (Pierce et al., 1989); Controls: gender, age, tenure			(Edwards & Lambert procedures)					

Table 2Sb. Study Outcomes

Study (Author, Year)	Country / Sector / Setting	Sample (n, population, demographics)	Study aims/hypothesis	Traits / Exposures (instrument, α)	Bullying Perpetration Outcomes (instrument, α, chronicity)	Other Variables (mediators, moderators, confounders)	Role / Target clarity	Design	Analysis	Effect Sizes (β / OR / r + 95% CI)	Effect Direction	Common-method risk	Context moderators	Replicability / Method Notes
	ing/engineering)	mean tenure 3.6 yrs; supervisors: 51% male,												
	Philippines / Banking sector (two large financi	66% aged 31+, 61.5% with ≥6 years tenure												

Table 2Sb. Study Outcomes

Study (Author, Year)	Country / Sector / Setting	Sample (n, population, demographics)	Study aims/hypothesis	Traits / Exposures (instrument, α)	Bullying Perpetration Outcomes (instrument, α, chronicity)	Other Variables (mediators, moderators, confounders)	Role / Target clarity	Design	Analysis	Effect Sizes (β / OR / r + 95% CI)	Effect Direction	Common-method risk	Context moderators	Replicability / Method Notes
	al institutions)													
Pullen & Rhodes (2008)*	UK; Leadership & Management; Case study in a	n = 1 (in-depth case study of a female leader, narrative identity work, textual materials)	To explore how narcissism, gender, and identity intersect in leaders' narratives; no formal hypothesis (exploratory, theory-building)	Narcissism (conceptual, psychoanalytic framing										

Table 2Sb. Study Outcomes

Study (Author, Year)	Country / Sector / Setting	Sample (n, population, demographics)	Study aims/hypothesis	Traits / Exposures (instrument, α)	Bullying Perpetration Outcomes (instrument, α, chronicity)	Other Variables (mediators, moderators, confounders)	Role / Target clarity	Design	Analysis	Effect Sizes (β / OR / r + 95% CI)	Effect Direction	Common-method risk	Context moderators	Replicability / Method Notes
	UK business school context													
Wislar et al. (2002)*	USA / Higher education (university emplo	Wave 1: n=2,492 (1,336 women, 1,156 men). Wave 2: n=2,038 (1,098 women, 940 men). Occupat	To examine whether personality vulnerabilities (neuroticism, narcissism) influence perceptions of sexual harassment (SH) and	Neuroticism and narcissism (short personality scales, internal reliabilities not reported in paper).	Not direct bullying perpetration. Outcomes were **perceived experiences**	Controlled for personality vulnerabilities (neuroticism, narcissism), demographics,	Role focus = victimization / exposure to SH and GWA (not perpetrators).	Two-wave longitudinal survey (1 year apart).	Logistic regression and OLS regression to predict drinking outcomes	ORs and betas reported narratively: SH and GWA significantly predicted deleterious drinking outcomes for both genders, even after controlling for personality. Effect sizes not given with CIs; magnitude described as "significant predictors."	ORs and betas reported narratively: SH and GWA significantly	Possible self-report bias, common-method variance.	Academic workplace context, occupational stratification.	Replicable in principle; two-wave design improves rigor, but effect sizes not fully reported limits meta-analysis usability.

Table 2Sb. Study Outcomes

Study (Author, Year)	Country / Sector / Setting	Sample (n, population, demographics)	Study aims/hypothesis	Traits / Exposures (instrument, α)	Bullying Perpetration Outcomes (instrument, α, chronicity)	Other Variables (mediators, moderators, confounders)	Role / Target clarity	Design	Analysis	Effect Sizes (β / OR / r + 95% CI)	Effect Direction	Common-method risk	Context moderators	Replicability / Method Notes
	yees)	ions included faculty, clerical, service/ maintenance, student workers/ trainees (RAs, TAs, residents, postdocs).	generalized workplace abuse (GWA), and whether these stressors predict deleterious drinking outcomes.		of SH and GWA (self-report, no α reported). Drinking outcomes: Michigan Alcoholism Screening Test (MAST);	occupation.			from SH/GWA and personality.		predicted deleterious drinking outcomes for both genders, even after controlling for personality.			

Table 2Sb. Study Outcomes

Study (Author, Year)	Country / Sector / Setting	Sample (n, population, demographics)	Study aims/hypothesis	Traits / Exposures (instrument, α)	Bullying Perpetration Outcomes (instrument, α, chronicity)	Other Variables (mediators, moderators, confounders)	Role / Target clarity	Design	Analysis	Effect Sizes (β / OR / r + 95% CI)	Effect Direction	Common-method risk	Context moderators	Replicability / Method Notes
					frequency of drinking to intoxication.						Effect sizes not given with CIs; magnitude described as “significant predictors.”			

[*] Not meta-eligible
