# Supplementary material for: Dark Triad traits and workplace bullying: a systematic review and meta-analysis of personality, power, and psychosocial safety
Source: Front Psychol. 2026 Mar 4;17:1738277. doi: 10.3389/fpsyg.2026.1738277 (PMC12995606; doi:10.3389/fpsyg.2026.1738277)
Supplement: Supplementary file 5 [file Data_Sheet_5.pdf]

Appendix Table S3. Study Quality -full ratings

Study (Author, Year)	CASP Tool (Cohort / Case-control / Qual / Other)	Clear aims (Y/N/CT)	Appropriate methodology/design (Y/N/CT)	Recruitment appropriate (Y/N/CT)	Exposure measurement valid/reliable (Y/N/CT)	Outcome measurement valid/reliable (Y/N/CT)	Confounding addressed (Y/N/CT) & key covariates	Statistical methods appropriate (Y/N/CT)	Missing data handled (Y/N/CT)	Ethical issues considered (Y/N/CT)	Findings clearly stated (Y/N/CT)	External validity / transferability (Y/N/CT)	Overall judgement (Low / Some / High)	Notes
Sheng et al., 2025.*	CASP Cohort (longitudinal survey)	Y	Y (time-lagged, multi-source, moderation analysis)	Y (hospital staff, though convenience sample)	Y (CWB scale validated; narcissism scale validated but α not reported)	Y (abusive supervision scale, α = .93)	Partial (controlled demographics, tenure; limited broader covariates)	Y (hierarchical regression; moderation tested with simple slopes; rigorous probing per Aiken & West, 1991)	Not clearly reported	Y (ethics approval mentioned)	Y (clear results, tables, figures)	Somewhat limited (single hospital, cultural specificity)	Some concerns (generalizability; limited confounder control)	Strengths: rigorous moderation probing ($\gamma = 4.30$, $p < .01$; simple slopes confirmed); validated scales; time-lag reduces CMV. Limitations :one hospital, gender-skewed sample, narcissism α not reported, unclear missing data handling.
Jang, Ki	CASP RCT/	Y	Y (appropriate	Y (volunte	Y (PNI validat	Y (NAQ-	Partial (no	Y (ANCOVA,	NR (s	Y (ethics	y	Limited (sing	Some con	Strengths: innovativ

Appendix Table S3. Study Quality -full ratings

**Study (Author, Year)**	**CASP Tool (Cohort / Case-control / Qual / Other)**	**Clear aims (Y/N/CT)**	**Appropriate methodology/design (Y/N/CT)**	**Recruitment appropriate (Y/N/CT)**	**Exposure measurement valid/reliable (Y/N/CT)**	**Outcome measurement valid/reliable (Y/N/CT)**	**Confounding addressed (Y/N/CT) & key covariates**	**Statistical methods appropriate (Y/N/CT)**	**Missing data handled (Y/N/CT)**	**Ethical issues considered (Y/N/CT)**	**Findings clearly stated (Y/N/CT)**	**External validity / transferability (Y/N/CT)**	**Overall judgement (Low / Some / High)**	**Notes**
m & Lee, 2025*	Quasi-experimental		for intervention)	ers, ICU nurses)	ed, Korean adaptation; α reported)	R/NAQ-P validated, adapted Korean)	stratified randomisation; small groups)	pre-post comparisons)	mall sample, some attrition risk)	approval reported)		le site, small N, gender imbalance)	concerns	e intervention, validated tools, ethics approval. Limitations: quasi-experimental, small sample, all-female intervention group, short follow-up
Braun et al., 2024 (Study 3)	Other (experimental event-recall; moderated medi	Y	Y	CT	Y	Y	Y (grandiose narcissism controlled; attributi	Y (PROCESS Model 8; bootstrapped CIs)	NR	Y ethics approved	Y	CT (leaders; sector/country NR)	Some concerns	Good sampling (supervisors, adequate Ns), validated measures, experimental manipula

Appendix Table S3. Study Quality -full ratings

Study (Author, Year)	CASP Tool (Cohort / Case-control / Qual / Other)	Clear aims (Y/N/CT)	Appropriate methodology/design (Y/N/CT)	Recruitment appropriate (Y/N/CT)	Exposure measurement valid/reliable (Y/N/CT)	Outcome measurement valid/reliable (Y/N/CT)	Confounding addressed (Y/N/CT) & key covariates	Statistical methods appropriate (Y/N/CT)	Missing data handled (Y/N/CT)	Ethical issues considered (Y/N/CT)	Findings clearly stated (Y/N/CT)	External validity / transferability (Y/N/CT)	Overall judgement (Low / Some / High)	Notes
	ation)						on conditions as moderators)							tions strengthen causal inference. Limitations: self-report risk, lower reliability for attribution measure in Study 1, short shame scale in Study 2. Strong internal validity; external validity limited by sample/context; self-report intentions.Overall

Appendix Table S3. Study Quality -full ratings

Study (Author, Year)	CASP Tool (Cohort / Case-control / Qual / Other)	Clear aims (Y/N/CT)	Appropriate methodology/design (Y/N/CT)	Recruitment appropriate (Y/N/CT)	Exposure measurement valid/reliable (Y/N/CT)	Outcome measurement valid/reliable (Y/N/CT)	Confounding addressed (Y/N/CT) & key covariates	Statistical methods appropriate (Y/N/CT)	Missing data handled (Y/N/CT)	Ethical issues considered (Y/N/CT)	Findings clearly stated (Y/N/CT)	External validity / transferability (Y/N/CT)	Overall judgement (Low / Some / High)	Notes
Jang et al., 2023	CASP – Analytical Cross-sectional / Other quantitative	Y – Explicit aim to test dark personality, perfectionism, mentalization → bullying perpetration/victimization among ICU nurses	Y – Cross-sectional survey with regression appropriate to aim	Y – Convenience online recruitment through hospital groupware & nurse communities; N = 416	Y – Dark Triad (Short Dark Triad, validated Korean version; α ≈ .80–.87); Perfectionistic Self-Presentation (validated; α reported in original)	Y – NAQ-R (victim) and NAQ-P (perpetrator); both validated, high α (.96 victim; .97 perpetrator)	Y – Controlled for organizational culture (α = .96), education, subjective health, etc.	Y – Multiple regression with diagnostics (VIF 1.04–3.62, Durbin–Watson 1.90, normality checked, Adjusted R^2 = .41)	CT – Missing data handling not explicitly described; final sample reported	Y – Ethical approval obtained; participants informed consent reported in methods	Y – Results tables and regression coefficients clearly presented	CT – Large, diverse ICU sample; but limited to South Korean tertiary hospitals; online convenience limits generaliz	Some concerns	: Moderate-to-high quality. Cross-sectional → cannot infer causality; self-report risk of bias; strong internal reliability; robust regression diagnostics; limited external validity beyond Korean ICU nurses

Appendix Table S3. Study Quality -full ratings

Study (Author, Year)	CASP Tool (Cohort / Case-control / Qual / Other)	Clear aims (Y/N/CT)	Appropriate methodology/design (Y/N/CT)	Recruitment appropriate (Y/N/CT)	Exposure measurement valid/reliable (Y/N/CT)	Outcome measurement valid/reliable (Y/N/CT)	Confounding addressed (Y/N/CT) & key covariates	Statistical methods appropriate (Y/N/CT)	Missing data handled (Y/N/CT)	Ethical issues considered (Y/N/CT)	Findings clearly stated (Y/N/CT)	External validity / transferability (Y/N/CT)	Overall judgement (Low / Some / High)	Notes
												ability		
Feng et al., 2023	CASP Cohort/ Other (quantitative, time-lagged, multilevel SEM)	Y– Explicit aims & hypotheses (Machiavellianism → LDG → abusive supervision; moderation by TMG; downstream team outcomes).	Y – Two-wave design, appropriate for testing mediation & moderation	Y– MTurk managers (Study 1) and intact teams in Chinese SOE (Study 2); large, relevant samples.	Y – Machiavellianism (Mach IV, α=.85 –.87); mediator/moderator scales with α>.90.	Y – Abusive supervision (Tepper, α=.95); subordinate-rated in Study 2 reduces bias.	Y– Controlled for demographics (leader age, gender, tenure, education, negative affectivity).	Y – Regression, bootstrapping, MSEM; appropriate for mediation/ moderation.	CT – Not deeply discussed; longitudinal attrition in Study 2 noted but not fully analy	Y– IRB approval, informed consent, voluntary participation mentioned.	Y – Clear tables, effect sizes, CIs reported	Y – Two cultures (US & China) improves generalisability; guanxi context limits transferability.	Some concerns – Mostly strong, but reliance on MTurk in Study 1 and guanxi-specific context may reduce generalisabi	Two large-sample, independent studies strengthen evidence. Excellent reliability & reporting. Minor concerns: attrition, CMV risk in Study 1, cultural specificity.

Appendix Table S3. Study Quality -full ratings

Study (Author, Year)	CASP Tool (Cohort / Case-control / Qual / Other)	Clear aims (Y/N/CT)	Appropriate methodology/design (Y/N/CT)	Recruitment appropriate (Y/N/CT)	Exposure measurement valid/reliable (Y/N/CT)	Outcome measurement valid/reliable (Y/N/CT)	Confounding addressed (Y/N/CT) & key covariates	Statistical methods appropriate (Y/N/CT)	Missing data handled (Y/N/CT)	Ethical issues considered (Y/N/CT)	Findings clearly stated (Y/N/CT)	External validity / transferability (Y/N/CT)	Overall judgement (Low / Some / High)	Notes
									sed				lity; missing data handling not fully detailed.	
Preston et al	CASP Other quantitative (cross-sectional survey)	Y– Aim clearly stated: test associations between psychopathic traits and workplace CWB	Y– Cross-sectional survey with regression appropriate to aim	Y– MTurk recruitment with inclusion criteria (≥100 HITs, ≥95% approval); atten	Y– Triarchic Psychopathy Measure (α=.87–.93), EPA Antisocial (α=.82)	Y – CWB-C (α=.96 total, .92–.94 subscales)	CT– Controlled for demographics; some Big Five controls in supplementary anal	Y– Hierarchical regression, correlations; interaction tests	CT – Missing data not discussed in detail; final n=331 aft	Y– IRB approval and informed consent reported	Y– Clear reporting of descriptive, regression results, and effect	CT – Online US adult workers; diverse occupations but MTurk limits	Some concerns	Strong reliability and appropriate statistics; but single-source cross-sectional design, MTurk sampling bias, limited info on missing data; external validity

Appendix Table S3. Study Quality -full ratings

Study (Author, Year)	CASP Tool (Cohort / Case-control / Qual / Other)	Clear aims (Y/N/CT)	Appropriate methodology/design (Y/N/CT)	Recruitment appropriate (Y/N/CT)	Exposure measurement valid/reliable (Y/N/CT)	Outcome measurement valid/reliable (Y/N/CT)	Confounding addressed (Y/N/CT) & key covariates	Statistical methods appropriate (Y/N/CT)	Missing data handled (Y/N/CT)	Ethical issues considered (Y/N/CT)	Findings clearly stated (Y/N/CT)	External validity / transferability (Y/N/CT)	Overall judgement (Low / Some / High)	Notes
				tion checks applied			ysis; not a full confounder model		er exclusions		sizes	generalisability		moderate. The main reasons for “Some concerns” rather than “Low risk”:Cross-sectional & single-source self-report → high common-method risk.Recruitment via MTurk → convenience, possible bias.Missing data handling not

Appendix Table S3. Study Quality -full ratings

Study (Author, Year)	CASP Tool (Cohort / Case-control / Qual / Other)	Clear aims (Y/N/CT)	Appropriate methodology/design (Y/N/CT)	Recruitment appropriate (Y/N/CT)	Exposure measurement valid/reliable (Y/N/CT)	Outcome measurement valid/reliable (Y/N/CT)	Confounding addressed (Y/N/CT) & key covariates	Statistical methods appropriate (Y/N/CT)	Missing data handled (Y/N/CT)	Ethical issues considered (Y/N/CT)	Findings clearly stated (Y/N/CT)	External validity / transferability (Y/N/CT)	Overall judgement (Low / Some / High)	Notes
														described in detail.
Fernández-del-Río et al., 2021	Other (quantitative cross-sectional survey)	Y – Aim to test incremental effect of Dark personality (incl. sadism) over Big Five on bullying perpetration (and victimization)	Y – Cross-sectional, hierarchical regression appropriate for incremental validity question	CT– Non-probability recruitment via university students distributing workplace surveys (broad sectors but convenience appr	Y – Standard Dark-trait and Big Five measures with internal consistency reported; Spanish versions used	Y– NAQ-Perpetrators (4 work-related items), validated tool; self-report perpetration focus	Y– Big Five included as covariates; demographics (age, gender, tenure) considered; stepwise models isolate incremental	Y– Hierarchical regression; ΔR^2 tests; correlations and reliabilities reported	Y– Listwise deletion: “after removing those with missing values … final n = 613”	CT– Voluntary, anonymity and confidentiality stated; explicit IRB/ethics board approval not clearly reported	Y– Results, coefficients, and conclusions clearly presented	CT– Spain-only, convenience sampling; good size/multi-sector but generalisability limited	Some concerns	Strengths: validated measures; clear perpetration outcome; robust regression and incremental validity; large n (613). Limitations: convenience sampling; self-report (CMV risk); cross-sectional (no causality); ethics approval

Appendix Table S3. Study Quality -full ratings

Study (Author, Year)	CASP Tool (Cohort / Case-control / Qual / Other)	Clear aims (Y/N/CT)	Appropriate methodology/design (Y/N/CT)	Recruitment appropriate (Y/N/CT)	Exposure measurement valid/reliable (Y/N/CT)	Outcome measurement valid/reliable (Y/N/CT)	Confounding addressed (Y/N/CT) & key covariates	Statistical methods appropriate (Y/N/CT)	Missing data handled (Y/N/CT)	Ethical issues considered (Y/N/CT)	Findings clearly stated (Y/N/CT)	External validity / transferability (Y/N/CT)	Overall judgement (Low / Some / High)	Notes
				oach)			variance							not explicitly stated though consent/anonymity described.
Priesemuth & Bigelow (2020)*	Other (quantitative, two field studies with Critical Incident Technique, regression & moderated mediation models)	Y – clear aim to test consequences of abusive supervision for perpetrators and moderating role of psychopathy	Y – CIT and time-lagged design appropriate for research question	CT – Student-mediated recruitment; mixed industries but convenience-based; risk of sampling bias	Y – Psychopathy measured with Triarchic Psychopathy Measure (TriPM), established reliability (α ≈ .84)	Y – Abusive supervision captured via CIT (self-reported incidents), validated coding approach	CT – Controlled for demographics (age, gender, tenure) but broader organizational covariat	Y – Regression, PROCESS mediation/moderation analyses appropriate	CT – Missing data handling not extensively reported; final N reduced but	Y – Consent obtained; voluntary participation stated; IRB approval noted	Y – Results presented clearly with tables & conditional effects	CT – Multi-sectoral (Canada, U.S.), but convenience recruitment limits generalisability	Some concerns	Strengths: multi-study design, use of CIT, multisource (Study 1) & time-lagged (Study 2), psychopathy moderation tested. Weaknesses: convenience sampling, unclear missing data

Appendix Table S3. Study Quality -full ratings

Study (Author, Year)	CASP Tool (Cohort / Case-control / Qual / Other)	Clear aims (Y/N/CT)	Appropriate methodology/design (Y/N/CT)	Recruitment appropriate (Y/N/CT)	Exposure measurement valid/reliable (Y/N/CT)	Outcome measurement valid/reliable (Y/N/CT)	Confounding addressed (Y/N/CT) & key covariates	Statistical methods appropriate (Y/N/CT)	Missing data handled (Y/N/CT)	Ethical issues considered (Y/N/CT)	Findings clearly stated (Y/N/CT)	External validity / transferability (Y/N/CT)	Overall judgement (Low / Some / High)	Notes
							es limited		no imputation described					handling, exposure – outcome mismatch for your review's meta focus.
Dåderman et al., 2019	CASP – Cross-sectional survey (Other)	Y	Y	CT (convenience sampling from municipalities, healthcare, schools)	Y (Dark Triad Dirty Dozen; validated; reliabilities reported)	Y (NAQ-R perpetrator version; α = .91)	Y (NAQ-R perpetrator version; α = .91)	CT – controlled for gender, age, education but limited covariates	N (no detailed handling of missing data described)	Y (ethics approval and consent procedures noted)	Y	CT (Swedish public sector sample; limits wider transferability)	Some concerns	Convenience sample and cross-sectional design limit representativeness; self-report for both predictors and outcomes increases CMV risk; otherwise strong instruments and

Appendix Table S3. Study Quality -full ratings

Study (Author, Year)	CASP Tool (Cohort / Case-control / Qual / Other)	Clear aims (Y/N/CT)	Appropriate methodology/design (Y/N/CT)	Recruitment appropriate (Y/N/CT)	Exposure measurement valid/reliable (Y/N/CT)	Outcome measurement valid/reliable (Y/N/CT)	Confounding addressed (Y/N/CT) & key covariates	Statistical methods appropriate (Y/N/CT)	Missing data handled (Y/N/CT)	Ethical issues considered (Y/N/CT)	Findings clearly stated (Y/N/CT)	External validity / transferability (Y/N/CT)	Overall judgement (Low / Some / High)	Notes
														transparent analysis. Strengths: Clear aims, appropriate design, validated measures (Dark Triad Dirty Dozen; NAQ-R), ethical approval, transparent regression analysis. Weaknesses: Convenience sampling (limits representativeness), no detailed missing data

Appendix Table S3. Study Quality -full ratings

Study (Author, Year)	CASP Tool (Cohort / Case-control / Qual / Other)	Clear aims (Y/N/CT)	Appropriate methodology/design (Y/N/CT)	Recruitment appropriate (Y/N/CT)	Exposure measurement valid/reliable (Y/N/CT)	Outcome measurement valid/reliable (Y/N/CT)	Confounding addressed (Y/N/CT) & key covariates	Statistical methods appropriate (Y/N/CT)	Missing data handled (Y/N/CT)	Ethical issues considered (Y/N/CT)	Findings clearly stated (Y/N/CT)	External validity / transferability (Y/N/CT)	Overall judgement (Low / Some / High)	Notes
														handling, modest confounder adjustment, self-report risk of CMV. Overall: Judged as "Some concerns" because of methodological limitations, though internal validity of measures is good.
Carré et al., 2018	CASP – Other (quantitative, cross-	Y – explicit aim to compare TriPM vs	Y – explicit aim to compare TriPM vs	CT – MTurk convenience sample;	Y – TriPM and SRP-SF validated; strong	Y – Workplace Deviance Scale and Sexual	Y – Controlled for demographic	Y – Controlled for demographics (gend	CT – Attention checks	Y – IRB approval and informed cons	Y – Results with βs, sig	CT – U.S.-only, MTurk convenie	Some concerns	Strengths: large sample (N = 481), validated measures,

Appendix Table S3. Study Quality -full ratings

Study (Author, Year)	CASP Tool (Cohort / Case-control / Qual / Other)	Clear aims (Y/N/CT)	Appropriate methodology/design (Y/N/CT)	Recruitment appropriate (Y/N/CT)	Exposure measurement valid/reliable (Y/N/CT)	Outcome measurement valid/reliable (Y/N/CT)	Confounding addressed (Y/N/CT) & key covariates	Statistical methods appropriate (Y/N/CT)	Missing data handled (Y/N/CT)	Ethical issues considered (Y/N/CT)	Findings clearly stated (Y/N/CT)	External validity / transferability (Y/N/CT)	Overall judgement (Low / Some / High)	Notes
	sectional survey)	SRP-SF for predicting workplace deviance & sexual harassment	SRP-SF for predicting workplace deviance & sexual harassment	diverse occupations but not representative	reliabilities (α = .70–.89)	Harassment Proclivity Scale validated, α = .88–.94	s (gender, age, race); tested incremental models	er, age, race); tested incremental models	used, but missing data handling not described beyond exclusions	ent reported	nificance, and interpretation clearly presented	nce sample; reduces generalisability		incremental validity test across two psychopathy models. Weaknesses: convenience MTurk recruitment, cross-sectional self-report (CMV risk), no longitudinal evidence, outcomes = self-reported proclivities not observed behaviours.

Appendix Table S3. Study Quality -full ratings

Study (Author, Year)	CASP Tool (Cohort / Case-control / Qual / Other)	Clear aims (Y/N/CT)	Appropriate methodology/design (Y/N/CT)	Recruitment appropriate (Y/N/CT)	Exposure measurement valid/reliable (Y/N/CT)	Outcome measurement valid/reliable (Y/N/CT)	Confounding addressed (Y/N/CT) & key covariates	Statistical methods appropriate (Y/N/CT)	Missing data handled (Y/N/CT)	Ethical issues considered (Y/N/CT)	Findings clearly stated (Y/N/CT)	External validity / transferability (Y/N/CT)	Overall judgement (Low / Some / High)	Notes
Pilch & Turska, 2015	CASP – Other (cross-sectional survey study)	Y – Aim clearly stated: role of Machiavellianism & organisational culture in bullying	Y – Cross-sectional survey appropriate for hypothesis testing	CT – Snowball sampling (limits representativeness, but adequate for exploratory aims)	Y – Mach-IV validated; α = .74	Y – NAQ perpetration/victimisation validated; α > .90	CT – Organisational culture included as moderator, but not all covariates (e.g., job stress, personality overlap)	Y – Hierarchical regression and moderation appropriate	N – Missing data handling not explicitly described	N – No explicit ethics approval/consent statement in article	Y – Results and effect sizes clearly presented	CT – Polish mixed-sector employees; generalisability limited beyond region and context	Some concerns	Strong instruments and clear results; snowball sampling and lack of ethics statement weaken robustness
Wang & Jia	Other (quantitative,	Y – Hypotheses stated	Y – Correlational/regre	CT – Convenience	Y – Narcissism measured	Y – Abusive supervision (	Y – Controlled s	Y – Controlled sex,	N/CT – Mi	CT – Consent/IRB	Y – Result	CT – Single city/	Some con	Cross-sectional, single-source

Appendix Table S3. Study Quality -full ratings

Study (Author, Year)	CASP Tool (Cohort / Case-control / Qual / Other)	Clear aims (Y/N/CT)	Appropriate methodology/design (Y/N/CT)	Recruitment appropriate (Y/N/CT)	Exposure measurement valid/reliable (Y/N/CT)	Outcome measurement valid/reliable (Y/N/CT)	Confounding addressed (Y/N/CT) & key covariates	Statistical methods appropriate (Y/N/CT)	Missing data handled (Y/N/CT)	Ethical issues considered (Y/N/CT)	Findings clearly stated (Y/N/CT)	External validity / transferability (Y/N/CT)	Overall judgement (Low / Some / High)	Notes
ng, 2014	cross-sectional survey)	(H1 narcissism ↓ perceived abuse; H2 abusive supervision → deviance, moderated by narcissism)	ssion design appropriate for moderation/mediation test	sample from an adult school in Beijing; participants from various companies; response rate NR	with short NPI (7 factors, 3 items each; CFA fit acceptable; α = .75)	Tepper 15-item, α = .87); Deviance toward supervisor (Mitchell & Ambrose 10-item, α = .83)	ex, age, tenure in regressions	age, tenure in regressions	ssing data handling not described; final N reported	not explicitly stated in the article	s and hypothesis tests reported clearly (r, R^2 change; interaction adds ~1% variance)	context; young sample (M age 26); limits generalisability	cerns	self-report (CMV risk); convenience sampling; ethics statement not explicit. Nevertheless, validated scales with good reliability, clear hypotheses and analyses. *Note for your review*: study models a busive supervision as predictor with narcissis

Appendix Table S3. Study Quality -full ratings

Study (Author, Year)	CASP Tool (Cohort / Case-control / Qual / Other)	Clear aims (Y/N/CT)	Appropriate methodology/design (Y/N/CT)	Recruitment appropriate (Y/N/CT)	Exposure measurement valid/reliable (Y/N/CT)	Outcome measurement valid/reliable (Y/N/CT)	Confounding addressed (Y/N/CT) & key covariates	Statistical methods appropriate (Y/N/CT)	Missing data handled (Y/N/CT)	Ethical issues considered (Y/N/CT)	Findings clearly stated (Y/N/CT)	External validity / transferability (Y/N/CT)	Overall judgement (Low / Some / High)	Notes
														m as moderator/mediator of deviance; it does not test trait → perpetration directly → not meta-eligible under your primary exposure – outcome spec.
Burton & Hoobler, 2011 *	Other (quantitative cross-sectional survey)	Y – Aim clearly stated: abusive supervision → aggression, mediat	Y– Moderated mediation survey design appropriate	CT– MBA students recruited with extra credit; cow	Y– Abusive supervision (Tepper 15-item, α = .91), narcissism	Y– Aggression outcome (adapted Bennett & Robinson scale,	Y– Aggression outcome (adapted Bennett & Rob	Y– Controlled for sex, age, tenure, negative affectivity	N– No explicit handling of missing	N/CT– No clear ethics approval/consent mentione	Y – Findings and effect sizes clearl	CT– Diverse industries, but limited to U.S. MBA	Some concerns	Strong psychometrics and analyses; major limitations: non-random MBA sample, lack of

Appendix Table S3. Study Quality -full ratings

Study (Author, Year)	CASP Tool (Cohort / Case-control / Qual / Other)	Clear aims (Y/N/CT)	Appropriate methodology/design (Y/N/CT)	Recruitment appropriate (Y/N/CT)	Exposure measurement valid/reliable (Y/N/CT)	Outcome measurement valid/reliable (Y/N/CT)	Confounding addressed (Y/N/CT) & key covariates	Statistical methods appropriate (Y/N/CT)	Missing data handled (Y/N/CT)	Ethical issues considered (Y/N/CT)	Findings clearly stated (Y/N/CT)	External validity / transferability (Y/N/CT)	Overall judgement (Low / Some / High)	Notes
		ed by interactional justice, moderated by narcissism		orkers invited via snowball; convenience sample	(NPI-16, α = .76), justice (Colquitt scale, α = .93)	α = .83)	inson scale, α = .83)		data described	d in article	y reported	working adults; convenience sampling limits generalisability		missing data handling, no ethics statement, potential CMV despite multi-source attempt.
Kiazad et al.	Cohort/observational (cross-sectional dyads) Cohort/observational (2-	Y	Y	Y (supervisor–subordinate dyads across multiple industries in Aust	Y (Mach IV scale, α = .76) Y (Mach IV scale, α = .74)	Y	Y	Y	CT CT	Y	Y	CT Y	Some concern Low concern	Cross-sectional design limits causal inference; self-report trait measure acceptable; strong reliability of outcome; mediatio

Appendix Table S3. Study Quality -full ratings

Study (Author, Year)	CASP Tool (Cohort / Case-control / Qual / Other)	Clear aims (Y/N/CT)	Appropriate methodology/design (Y/N/CT)	Recruitment appropriate (Y/N/CT)	Exposure measurement valid/reliable (Y/N/CT)	Outcome measurement valid/reliable (Y/N/CT)	Confounding addressed (Y/N/CT) & key covariates	Statistical methods appropriate (Y/N/CT)	Missing data handled (Y/N/CT)	Ethical issues considered (Y/N/CT)	Findings clearly stated (Y/N/CT)	External validity / transferability (Y/N/CT)	Overall judgement (Low / Some / High)	Notes
	wave longitudinal dyads)			ralia) dyads from 2 Philippine banks; clear recruitment)										n tested carefully Time-lag reduces common method bias; moderator (OBSE) strengthens theoretical test; well-replicated across cultures
Pullen & Rhodes (2008)*	CASP Qualitative	Y – explicit focus on gendered narcissism & identity work in leadership	Y – narrative, critical case study design appropriate	Y – narrative, critical case study design appropriate	CT – single case, not “recruitment” in quantitative sense	CT – constructs interpreted via psychoanalytic/narrative theory, no standardised scales	CT – outcomes conceptualised (identity, power, lead	N – confounding not applicable (interpretive, not causal analysis)	CT – no statistics; interpretive textual an	CT – ethics not discussed in detail, though anonymisation implied	Y – rich, theoretically grounded findings clear	CT – limited transferability (single case), but conceptual	Some concerns – strong theory-building, but limited emp	Reflexive, interpretive qualitative study. Strong in depth/theory, weak in breadth/generalisation. Not meta-analysis

Appendix Table S3. Study Quality -full ratings

Study (Author, Year)	CASP Tool (Cohort / Case-control / Qual / Other)	Clear aims (Y/N/CT)	Appropriate methodology/design (Y/N/CT)	Recruitment appropriate (Y/N/CT)	Exposure measurement valid/reliable (Y/N/CT)	Outcome measurement valid/reliable (Y/N/CT)	Confounding addressed (Y/N/CT) & key covariates	Statistical methods appropriate (Y/N/CT)	Missing data handled (Y/N/CT)	Ethical issues considered (Y/N/CT)	Findings clearly stated (Y/N/CT)	External validity / transferability (Y/N/CT)	Overall judgement (Low / Some / High)	Notes
							ership practice), not measured with instruments		alysis applied rigorously		y presented	contribution strong	irical generalisability	eligible but may inform conceptual framing.
Wislar et al., 2002*	Cohort (two-wave longitudinal workplace survey)	Y	Y	Y	Y(university employees, sufficient size)	(university employees, sufficient size)	Y (harassment and abuse experiences self-reported, psychometrically sound)	CT – controlled for personality traits but limited other covariates	Y (appropriate regression analyses)	CT (no detailed ethical statement, but implied IRB approval)	Y	CT (academic setting limits wider generalisability)	Some concerns	Longitudinal design and validated measures strengthen results, but limited reporting on missing data and ethics reduces transparency. Outcomes are more

Appendix Table S3. Study Quality -full ratings

Study (Author, Year)	CASP Tool (Cohort / Case-control / Qual / Other)	Clear aims (Y/N/CT)	Appropriate methodology/design (Y/N/CT)	Recruitment appropriate (Y/N/CT)	Exposure measurement valid/reliable (Y/N/CT)	Outcome measurement valid/reliable (Y/N/CT)	Confounding addressed (Y/N/CT) & key covariates	Statistical methods appropriate (Y/N/CT)	Missing data handled (Y/N/CT)	Ethical issues considered (Y/N/CT)	Findings clearly stated (Y/N/CT)	External validity / transferability (Y/N/CT)	Overall judgement (Low / Some / High)	Notes
														focused on harassment/abuse & drinking, not pure bullying perpetration.
